# Ecological Stability Properties of Microbial Communities Assessed by Flow Cytometry

**DOI:** 10.1128/mSphere.00564-17

**Published:** 2018-01-17

**Authors:** Zishu Liu, Nicolas Cichocki, Fabian Bonk, Susanne Günther, Florian Schattenberg, Hauke Harms, Florian Centler, Susann Müller

**Affiliations:** a Department of Environmental Microbiology, UFZ-Helmholtz Centre for Environmental Research, Leipzig, Germany; University of Iowa

**Keywords:** constancy, microbial communities, microbial ecology, microbial flow cytometry, resilience, resistance, single-cell analytics, stability properties

## Abstract

Natural microbial communities affect human life in countless ways, ranging from global biogeochemical cycles to the treatment of wastewater and health via the human microbiome. In order to probe, monitor, and eventually control these communities, fast detection and evaluation methods are required. In order to facilitate rapid community analysis and monitor a community’s dynamic behavior with high resolution, we here apply community flow cytometry, which provides single-cell-based high-dimensional data characterizing communities with high acuity over time. To interpret time series data, we draw inspiration from macroecology, in which a rich set of concepts has been developed for describing population dynamics. We focus on the stability paradigm as a promising candidate to interpret such data in an intuitive and actionable way and present a rapid workflow to monitor stability properties of complex microbial ecosystems. Based on single-cell data, we compute the stability properties resistance, resilience, displacement speed, and elasticity. For resilience, we also introduce a method which can be implemented for continuous online community monitoring. The proposed workflow was tested in a long-term continuous reactor experiment employing both an artificial and a complex microbial community, which were exposed to identical short-term disturbances. The computed stability properties uncovered the superior stability of the complex community and demonstrated the global applicability of the protocol to any microbiome. The workflow is able to support high temporal sample densities below bacterial generation times. This may provide new opportunities to unravel unknown ecological paradigms of natural microbial communities, with applications to environmental, biotechnological, and health-related microbiomes.

**IMPORTANCE** Microbial communities drive many processes which affect human well-being directly, as in the human microbiome, or indirectly, as in natural environments or in biotechnological applications. Due to their complexity, their dynamics over time is difficult to monitor, and current sequence-based approaches are limited with respect to the temporal resolution. However, in order to eventually control microbial community dynamics, monitoring schemes of high temporal resolution are required. Flow cytometry provides single-cell-based data in the required temporal resolution, and we here use such data to compute stability properties as easy to interpret univariate indicators of microbial community dynamics. Such monitoring tools will allow for a fast, continuous, and cost-effective screening of stability states of microbiomes. Applicable to various environments, including bioreactors, surface water, and the human body, it will contribute to the development of control schemes to manipulate microbial community structures and performances.

## Abstract (precis)

Microbial communities drive many processes which affect human well-being directly, as in the human microbiome, or indirectly, as in natural environments or in biotechnological applications. Due to their complexity, their dynamics over time is difficult to monitor, and current sequence-based approaches are limited with respect to the temporal resolution. However, in order to eventually control microbial community dynamics, monitoring schemes of high temporal resolution are required. Flow cytometry provides single-cell-based data in the required temporal resolution, and we here use such data to compute stability properties as easy to interpret univariate indicators of microbial community dynamics. Such monitoring tools will allow for a fast, continuous, and cost-effective screening of stability states of microbiomes. Applicable to various environments, including bioreactors, surface water, and the human body, it will contribute to the development of control schemes to manipulate microbial community structures and performances.

## INTRODUCTION

Natural microbial communities (NMCs) drive most biogeochemical cycles, are associates of higher organisms (as in the human microbiome), and are essential catalysts in many biotechnological processes. In recent years, new molecular tools have allowed us to resolve the compositions and the functional repertoires of NMCs. While the human microbiome is often the focus (1–3), NMCs are also interesting for biotechnological applications, as complementary abilities of community members, for example, those used for substrate oxidation, can reduce the need for genetically engineered pure strains (4). Additionally, functional redundancy within the community can improve process stability. In health and biotechnology, the idea of controlling NMCs (e.g., by medication or augmentation) has been raised. However, such efforts are currently based solely on experience due to the lack of a proper understanding of NMC dynamics.

To fill this gap, macroecological concepts are increasingly applied to NMCs (5–7), including diversity (8, 9), community function (10, 11), and trait distributions (12, 13), which are contributing factors for community stability in all natural and artificial ecosystems (14, 15). In macroecology, the term “stability” is widely discussed in a more general way, even with socioecological systems (16), but it can also be used to more specifically describe single mechanisms operating on specific biological levels (17). In this paper, we build on macroecological theories to develop a concept of stability that can be used in a workflow to monitor and eventually adjust microbial communities in artificial, human, and natural environments.

The discussion of stability in ecological theory has led to a myriad of partially overlapping definitions which cover different aspects of stability. In response to this confusion, Grimm and Wissel (18) have distilled the essence of this discussion, identifying six main stability properties: constancy, resilience (RL), persistence, resistance (RS), elasticity (E), and domain of attraction. “Constancy” refers to the ability of a system to stay “essentially unchanged.” “Resilience” describes the ability of a system to return to its prior state after a temporary disturbance-induced state change (18). “Persistence,” in turn, refers to the ability of an ecological system to last as an identifiable entity throughout time (18). “Resistance” describes the ability of a system to stay unchanged despite disturbances, and “elasticity” refers to the time span that a system needs to return to its original state after a disturbance (18). Finally, the “domain of attraction” subsumes all postdisturbance states from which the original state can be reached again. Applying these concepts to assess how human or environmental NMCs respond to and recover from arbitrary disturbance events, we require NMCs to be in a state of constancy. This allows us to define a predisturbance reference state (*s*_ref_) as the basis for our calculations. To quantify NMC alterations, we propose a new protocol to calculate the properties resistance, resilience, and elasticity. Additionally, we introduce the property displacement speed (DS), which, by analogy to elasticity, describes how long it takes following a disturbance event for the system to reach its disturbed state. Although the domain of attraction is computable if the system can be described by a mathematical model, it is difficult to define for natural systems, such as the human microbiome. Also, the property persistence will not be considered in this study because we do not consider the extinction of whole microbiomes. Our new workflow uses the reductionist stability definitions as suggested by Grimm and Wissel (18) and does not refer to the more holistic view on resilience suggested by Holling, which also includes socioecological aspects (19).

Quantifying four stability properties, we want to contribute to an understanding of microbiome-driven processes by introducing the concept of community stability as a means of developing community adjustment and control strategies. Differently from what has been developed so far for sequence-based approaches (see, e.g., reference 20), we use individual-based information for our concept. The cytometric analysis of microbial communities relies on metadata and provides morphological and physiological information on single cells, as well as cell abundance quantities within microbial communities (21–23). We here focus on compositional stability, in contrast to functional stability, as the former is likely to facilitate the latter. We note however, that due to functional redundancies in NMCs, functional stability may not require compositional stability. Recently, bioinformatics tools which allow for an automatic evaluation were provided for cytometric data sets (23, 24). The benefit of cytometric data for microbial ecological research is the high sample density per time period and between locations and the availability of data in nearly real time. First attempts were made by Koch et al. (25), and only recently, this monitoring approach successfully identified species-sorting and mass-transfer paradigms as the dominant mechanisms shaping wastewater meta-communities (26). In addition, a phenotypic-diversity metric that allows for the study of ecological succession of *in situ* microbial communities is now available (27).

In this study, we want to expand the cytometric ecological toolbox further by providing a mathematical background and a step-by-step workflow to calculate stability properties of complex communities. In order to demonstrate the applicability of our workflow, both an artificial and a complex community were grown under steady-state conditions. Community stability was debalanced by short-term alterations of physicochemical conditions. Samples were also subjected to Illumina sequencing for confirmation of cytometric data.

## RESULTS

To demonstrate the usefulness of our workflow for assessing ecological stability properties of microbial communities (see Materials and Methods), we exemplarily applied it to a long-term continuous reactor experiment in which, after prolonged cultivation, an artificial microbial community (AMC) of low complexity was amended with a complex microbial community (CMC) of high complexity. To mimic disturbances, pH and temperature were altered for short time periods. The various structures of the AMC and CMC were monitored by flow cytometry at the single-cell level, the resulting data were evaluated using the tool flowCyBar (see Materials and Methods), and stability properties were calculated according to the proposed novel workflow.

### Ecological situation.

As proposed by Grimm and Wissel (18), first the ecological situation must be defined before stability properties can be calculated. Of the six features, the first three are the same for any stability property assessment based on flow cytometric data (see Materials and Methods), while the remaining three are application specific. For completeness, we list all six features for our reactor experiment: (i) the level of description refers to the whole microbial community; (ii) the variable of interest is the community structure (relative abundances of gate populations); (iii) for defining the reference state, we took the last sample prior to the respective disturbance event; technical replicates had a mean standard deviation of gate abundances of 0.6% [see Text S2, section S2.3, in the supplemental material] and led to a value of 0.16 for the radius defining the reference space size [*r*_*c*_] using the Canberra distance; (iv) as the disturbance, a continuous reactor system was consecutively subjected to five disturbance events (the continuous reactor was started with a low-diversity AMC, which, once established, was treated with short-term pulses of alterations in temperature and pH; subsequently, a more diverse CMC was added, and again, temperature and pH were changed for short time periods [2 to 11 h]); (v) regarding the temporal scale, the reactor was operated continuously for 435 h, with the AMC cultivated for 216 h and the CMC cultivated for 219 h; the time between disturbances ranged from 39 h to 117 h; and (vi) regarding the spatial scale, the reactor was well mixed, and hence, space was neglected in the system.

### Stability properties during steady-state operation.

**FIG 1:**
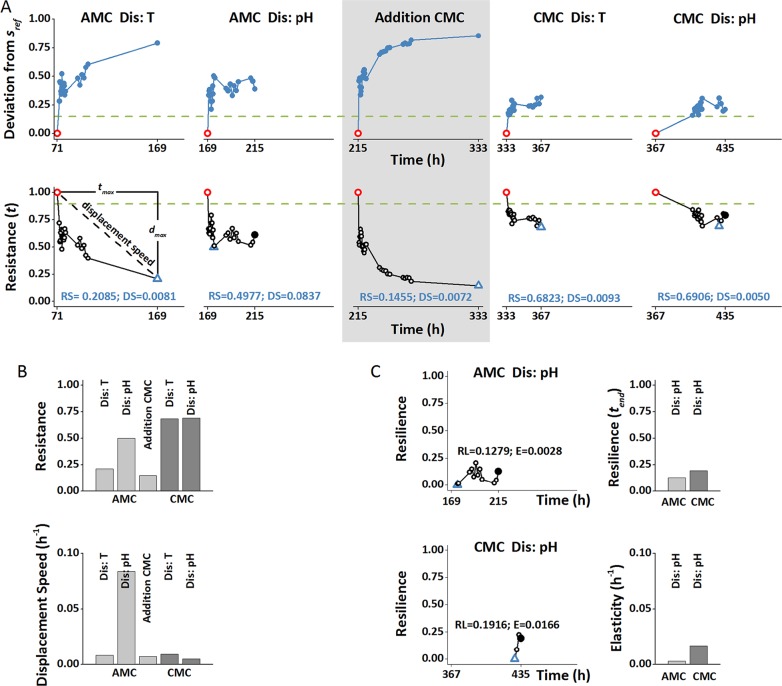
Response of microbial communities to disturbance events and stability properties of resistance, displacement speed, resilience, and elasticity (Table 1). A low-complexity member community (AMC) and, after addition (gray box), a complex community (CMC) were cultivated under the same conditions. Both the AMC and CMC structures were displaced in response to short-term temperature and pH disturbances (Dis: T and Dis: pH, respectively). (A) Deviation from the reference state (*s*_ref_) (red circle), calculated as the Canberra distance, and resistance over time. Dashed horizontal green lines indicate the border of the reference space, blue triangles mark *s*_max_, and filled black circles mark *s*_end_. The determination of resistance (RS) and displacement speed (DS) is shown as a dashed black line. (B) Comparison of the stability properties resistance and displacement speed across all disturbance experiments. (C) Comparison of the stability properties resilience and elasticity for disturbance experiments showing resilience (AMC Dis: pH and CMC Dis: pH).

Calculating stability properties as proposed by the workflow showed that all disturbance events led to immediate strong changes in both AMC and CMC (Fig. 1A), indicating that both communities were affected by these disturbances. The magnitudes of disturbance-induced shifts were different for the community types. CMCs showed the strongest resistance with very similar RS values of 0.68 and 0.69 for the temperature and pH disturbances, respectively (Fig. 1B), even though an unintentional transient oxygen deficiency (400 h) occurred shortly before the second pH disturbance. Hence, the observed RS value following this additional disturbance might represent a mixed response. AMCs were more affected by disturbances, and their responses differed more between disturbance types. Highest resistance was detected for the pH disturbance with an RS value of 0.50 (Fig. 1B). The temperature disturbance had a stronger impact, resulting in a lower resistance, with an RS value of 0.21. As expected, the lowest resistance was measured for the disturbance resulting from the introduction of the CMC into the continuous reactor, which changed the AMC structure within 26 h (Text S2, section S2.7). An RS value of 0.15 marked this least-resistant case in our experiment. Except after the pH disturbance with the AMC, displacement speed values were similar and ranged from 0.005 to 0.009 h^−1^ (Fig. 1B). For these experiments, the maximal deviation (*d*_max_) was reached at the end, or close to the end, of the experiment (*t*_end_). This was not the case for the pH experiment using the AMC, where the maximal deviation was reached early on, leading to a higher displacement speed of 0.084 h^−1^.

In no case was the reference space reached again at the end of the experiment. However, although in the temperature disturbance experiments and the CMC inoculation experiment, the distance tended to continuously increase over time, in the pH disturbance experiments, this trend was reversed after an initial phase of increasing distance. For these two experiments, resilient behavior was observed (Fig. 1C). Resilience values of 0.13 for the AMC and 0.19 for the CMC indicated, however, that recovery was far from complete, with the CMC showing a better recovery.

Elasticity values could be determined only for the pH perturbations for which resilient behavior was observed (Fig. 1C). Elasticity was higher for the CMC (E = 0.017 h^−1^) than for AMC (E = 0.0028 h^−1^), indicating a faster recovery of the CMC.

Overall, the CMC was less affected by the disturbances than the AMC, as evidenced by higher resistance, resilience, and elasticity values (Fig. 1B and C). Values for displacement speeds were in the same range for the AMC and CMC except in the pH disturbance experiment using the AMC, where the maximal deviation was reached much sooner than in all other cases (Fig. 1B).

While the proposed analysis so far depends on the knowledge of the maximal deviation, which can be known only *post hoc*, a modified version of the resilience equation as described in the workflow (Materials and Methods) in combination with the current deviation from the reference state [*d*(*t*)] can also be used to monitor NMC dynamics in an online fashion, once a reference state is defined. Online resilience can unambiguously identify phases in which the system is pushed further away from the reference state (indicated by a resilience value of 0) from phases in which it approaches the reference state again (values >0). It also quantifies the success of recovery, with a value of 1 indicating the perfect return to the reference state and lower values indicating partial recovery. Online resilience cannot, however, quantify the severity of phases in which the deviation increases. This in turn is directly observable from the current deviation *d*(*t*). Hence, both properties should be used in tandem, providing a powerful means for online monitoring able to detect stress and recovery phases of complex microbial communities.

## DISCUSSION

Macroecological concepts to reveal underlying paradigms of microbial community behavior can be most valuable in the many areas where microorganisms are important mediators of biotechnological and biogeochemical processes. However, the use of these ecological concepts in microbiology is currently limited to the calculation of diversity properties and is frequently based on next-generation sequencing (NGS) data. NGS, however, is still too expensive and time-consuming in the handling, evaluation, and calculation of sample data to be applied at frequencies similar to microbial generation times, which is a necessity when fast changes in community composition need to be detected. Although we used 16S rRNA gene amplicon sequencing in this study to underpin our cytometric data, it became clear that sequencing-based approaches are of limited utility for tracking and being able to respond to rapid changes in community structures. Instead, cytometry provides multivariate data sets for single cells that indicate community structure changes quickly and inexpensively. Variation in any microbial community structure is always caused by changes in either intrinsic population characteristics (cell numbers and types) or/and abiotic data (e.g., pH or temperature). Thus, the step from monitoring and observing microbial community structure shifts to understanding of their biotic or abiotic causes would help to realize control of human, managed, or natural ecosystems. In the last few years, rapid and easy-to-use tools were established to analyze and visualize microbial community dynamics (based on single-cell analytics) in a nearly fully automated fashion (23, 28). Recently, and similarly to how data are exploited by NGS-based technologies in microbiology, ecosystem concepts were implemented in single-cell-based workflows showing that α, β, and γ diversities can be revealed using evenness and richness properties for diversity calculations based on bins (27) or gates (26) (parts of both approaches were applied to the data in this study, and results are shown in Text S6 in the supplemental material). While bin-based data usually do not need to be processed prior to further calculations and evaluation, the gate-based approach requires a gate template, which comprises the most abundant cell clusters of a community, to be defined. Both approaches have advantages and disadvantages. Bin-based evaluations need high computational power when time-dependent data analysis is required and bead information used for standardization of cytometric analysis must be removed. Gate-based computing and evaluation procedures concentrate on the most important structures of a community (e.g., by MultiCola subtraction [26, 29]), enabling sorting and easy-to-use visualization techniques, such as flow CyBar (23), but they need the creation of a gate template either *post hoc* or “atline” (i.e., in such a fashion that analysis results are rapidly available for process control). For our workflow, we decided to use the gate-based computation and evaluation approach. We note, however, that our approach is equally applicable to a bin-based evaluation scheme in which gates simply need to be replaced by bins.

We based our workflow on the stability concept predominantly to unravel stability properties of engineered and natural microbial communities in confined environments, such as the human microbiome, which can easily be monitored by single-cell analytics (30). For the first time, we here propose a method to quantify crucial stability properties based on single-cell data. These properties were taken from the macroecology concept of Grimm and Wissel (18) and paired to the properties resistance (RS) and displacement speed (DS), as well as resilience (RL) and elasticity (E) (Table 1). We created a workflow that, using these four properties, characterizes the degrees and kinds of responses of microbial communities to disturbances. For easy and fast application of our workflow, we provide an R script requiring minimal user input (yet being highly configurable). It computes numerical values for the stability properties discussed and generates graphical visualizations of community dynamics in response to disturbance events based on time series data of relative gate abundances (Text S4).

**TABLE 1:**
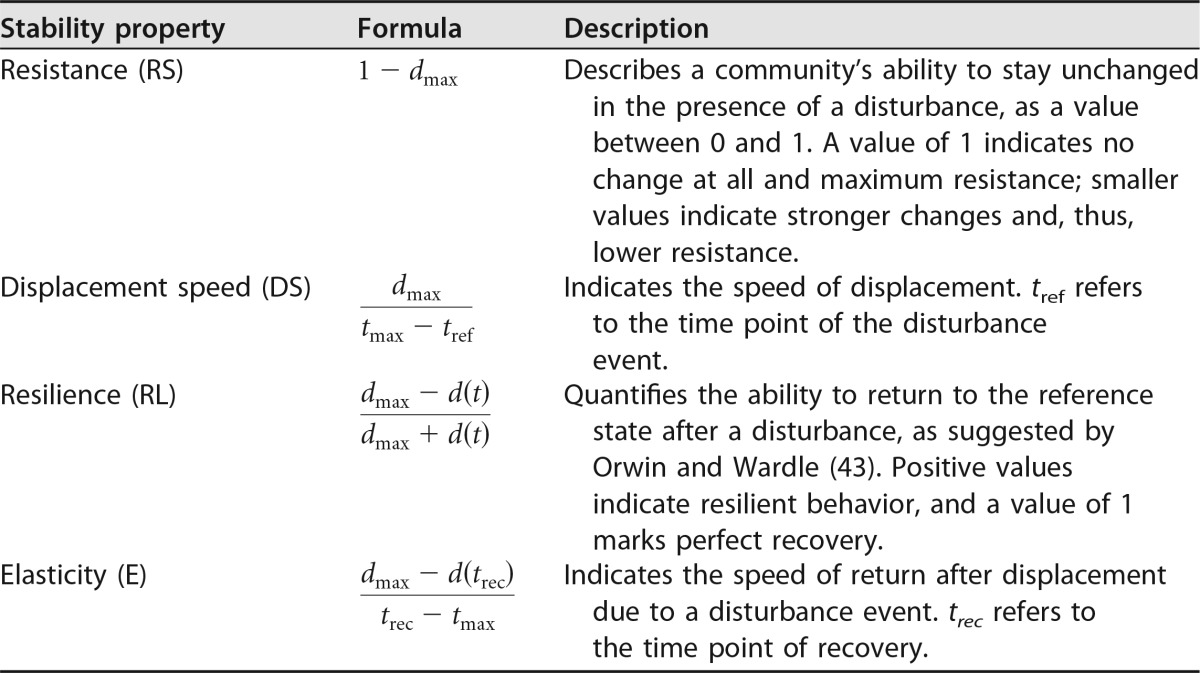
Calculation of stability properties describing displacement and recovery of the system by the disturbance (see Table 2 for definitions of terms)

When applying our concept to two communities of low (AMC) and high (CMC) structure complexities, we were able to infer different stability properties from responses to disturbances which were intentionally caused by pH and temperature variations. All other parameters were stable due to steady-state conditions, and also cell count values showed plateaus for the AMC (at ~2 × 10^8^ cells ⋅ ml^−1^) and CMC (at ~1 × 10^10^ cells ⋅ ml^−1^). However, community structure was clearly differently affected by the type of the disturbances (pH versus temperature) and the complexity of the community (AMC versus CMC), which caused clear and distinct trends in their evolutions. The more complex community acted generally with higher resistance and resilience. That complex communities show higher stability properties seems common and is often described both in macroecology (31, 32) and in microbial ecology (33, 34). Both communities showed no resilience upon temperature disturbance, whereas the pH disturbance was obviously less severe since the CMC showed high resilience and elasticity values and even the AMC recovered from this type of disturbance, although to a clearly lesser extent. Although a full interpretation of the biological implications of our findings regarding our exemplary microbiomes is beyond the scope of this work, we assume that the decrease in temperature favored the *Bacillus* strain over the other two organisms probably because of its protection from cold shock proteins (35). The *Bacillus* strain indeed became dominant, as was verified by the sequencing analysis (Text S5, section S5.4).

The methodology for calculating our four stability properties requires a deviation from a reference state (*s*_ref_). This in turn requires a suitable reference state to exist, demanding constant community states prior to the disturbance event, referring to a constant structure. To properly define the reference space, the experiment should include a suitably long, undisturbed phase prior to the disturbance in which representative reference samples can be obtained. In our test sample, the mean natural variability in the reference state (σ_ref_) was 0.6%, which is a low value (see the technical deviation in Text S2, section S2.3). By using natural communities, earlier studies determined much higher values of 25% for intrinsic community variations (36), and in macroecology, values as high as 37% have been described (42). We note that if predisturbance community dynamics are governed by Lotka-Volterra-type regular oscillations (37–39), the reference space would become very large. In this case, although we can detect whether an oscillatory system returns to its initial state range, we are not able to distinguish a return to its original regular oscillatory dynamics from a return to random fluctuations coincidentally covering the same range. For such systems, metrics that explicitly consider temporal dynamics are more suitable (see, e.g., reference 40).

When the reference state is defined, deviations from this state are used for the quantitative calculation of stability properties of a microbial community. Both the Euclidean distance and the Canberra distance showed similar trends (Fig. S4.1 in Text S4), although we noted that the Canberra distance is generally more suitable and is thus recommended for the workflow (see Materials and Methods). For the calculated stability properties resistance and resilience, values within a well-defined numerical range are provided by the workflow, allowing for easy comparability between different experimental situations or even between localities. More care must be taken when interpreting values of the stability properties displacement speed and elasticity. Both refer to a speed for which no predefined value range exists. Especially when using these two stability properties to compare different localities, the typical time scales of these systems must also be considered, including, for example, generation times of organisms or hydraulic retention times (which can refer to bioreactors but also to the different parts of the human digestive system). In our experimental setting, this was of no concern, as the operating regime of the continuous reactor, including dilution rate, was not altered throughout the experiment.

Tools for online monitoring and evaluation, as was demonstrated by the proposed equation to compute online resilience (see Materials and Methods), are highly desirable to immediately interpret microbial community dynamics in order to develop strategies for their adjustment and control. The workflow is applicable to future research which may address microbial community design, treatment, and operation. Our online stability tool might not answer the question of which organisms in a community fail to function or are about to go extinct, but it is able to constantly monitor community structure variations by determining RL values. Phylotypes or specific cell functions can nevertheless always be determined atline by cell sorting of selected subcommunities and subsequent sequencing, as was shown for our sample experiment (Text S2, section S2.1, and Text S5).

In conclusion, we have presented a workflow for quantifying the stability properties resistance, resilience, displacement speed, and elasticity, originally derived from the field of macroecology and adopted here for microbial communities. Instead of tracking changes in function, we focused on tracking changes in community structure, which is more informative, as a structural change may precede functional changes, hence providing an early warning signal. We used single-cell phenotypic data and showed that our computational methods clearly characterize and quantify a microbial community’s responses to disturbances in a confined microbial environment. Relying on the rapidity of data generation and their bioinformatics evaluation, we additionally introduced a procedure that allowed for an online computation of resilience. The presented workflow based on stability properties introduces a novel strategy for monitoring natural microbial communities in human, managed, and natural environments, either *post hoc* or atline, and is much faster than state-of-the-art methods based on sequence data.

## MATERIALS AND METHODS

### Experimental design.

To exemplify the computation and interpretation of our proposed stability properties, a continuous reactor experiment exposing microbial communities to defined disturbances was performed. The continuous reactor provided a balanced situation for studying the dynamics of both an artificial microbial community (AMC) and a complex microbial community (CMC), derived from a full-scale wastewater treatment plant, during the course of a long experiment (435 h) (see Text S1 in the supplemental material for details). Thus, we generated community states that can be assumed to be well balanced and which served effectively as reference states for transient (2- to 11-h) disturbances. Only one long-term disturbance was initiated by the addition of the CMC after cultivating the AMC for 216 h. The short-term pulse disturbances were caused by pH and temperature alterations, which were applied to both communities. We assume that the reactor content is perfectly mixed so that spatial variation does not play any role. The continuous reactor was run in a way that excluded unwitting recruitment of species, thus representing an isolated patch location. Both the biotic parameter cell number (Fig. S1.1A in Text S1) and the abiotic parameters dilution rate (per hour), aeration rate (liters per minute), temperature (degrees Celsius), and pH (Fig. S1.1B in Text S1) were recorded during the experiment.

10.1128/mSphere.00564-17.1 TEXT S1 Bacterial strains and culture conditions. Download TEXT S1, PDF file, 0.3 MB. Copyright © 2018 Liu et al. 2018 Liu et al. This content is distributed under the terms of the Creative Commons Attribution 4.0 International license.

10.1128/mSphere.00564-17.2 TEXT S2 Flow cytometric analysis. Download TEXT S2, PDF file, 1.7 MB. Copyright © 2018 Liu et al. 2018 Liu et al. This content is distributed under the terms of the Creative Commons Attribution 4.0 International license.

### Flow cytometric analysis.

Harvested, fixed, and DNA-stained samples were measured with a MoFlo Legacy cell sorter. The procedures for cell number determination and cell sorting are outlined in Text S2 (section S2.1), accompanied by an overview of cytometric terms used (section S2.2). The degree of deviation between technical replicates was determined to be 0.6% by calculating abundances of cells in gates (expressed in percentages) and their mean standard deviation (Text S2, section S2.3).

### Cytometric evaluation tools.

**FIG 2:**
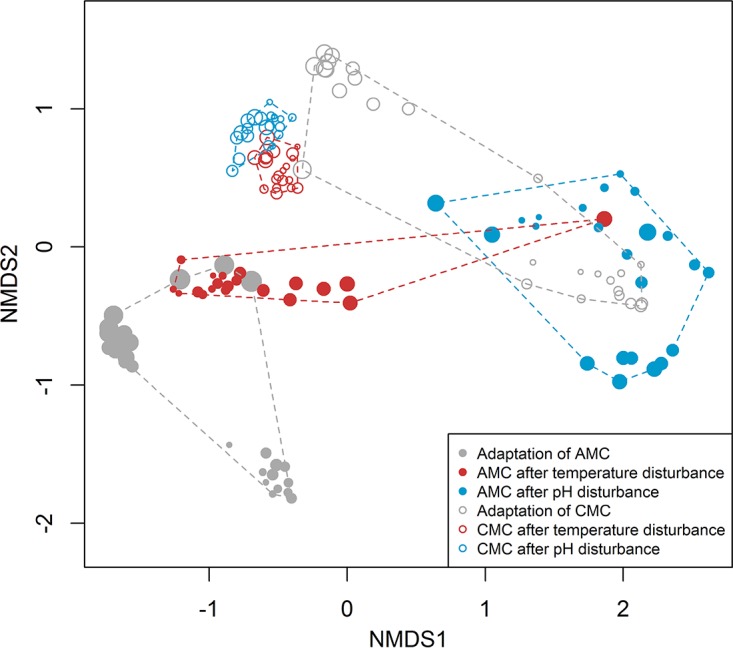
Community dynamics over the full experiment. Nonmetric multidimensional scaling (NMDS) plot (Bray-Curtis dissimilarity) indicating community dynamics of the artificial microbial community (AMC) (0 to 215 h, filled circles) and the complex microbial community (CMC) (216 to 435 h, open circles). Symbol size increases with increasing sampling time.

Cytometric data were acquired with Summit version 4.3 (Beckman Coulter, Brea, CA) and FlowJo V10 (FlowJo, LLC, OR, USA). Usually, information on cell size (forward scatter [FSC]) and chromosome contents (DAPI [4′,6-diamidino-2-phenylindole] fluorescence) were recorded together and visualized in a two-dimensional (2D) dot plot. Two hundred fifty thousand cells were analyzed per measurement within the parent gate (Fig. S2.3 in Text S2) and distributed virtually according to those characteristics. Subclusters of cells with different characteristics were recorded by setting gates and creating a gate template (Text S2, section S2.4). Thus, within one gate, we collected cells that represent similar characteristics with regard to cell size and DNA contents. The compositions of samples were received by evaluating the positions of gates in a histogram and calculating the numbers of cells per gate (all values are given in Text S2, section S2.5). The gate template defined a total of 34 gates (with noise and beads removed) whose relative abundances ranged between 0% (gate 8, 19 h) and a maximum of 72.3% (gate 21, 175.5 h), with an average fraction of cells per gate of 2.94%. The variation in samples over time is recorded in a movie (Movie S1) and shown for exemplary samples (Text S2, section S2.6), and values were calculated by using the bioinformatic tool flowCyBar (Text S2, section S2.7; http://www.bioconductor.org/packages/release/bioc/html/flowCyBar.html [23, 28]). The data clearly show how the community changes in response to the applied perturbations in the bioreactor (Fig. 2).

10.1128/mSphere.00564-17.7 MOVIE S1 QuickTime movie showing the flow cytometric dot plot (FSC and DAPI fluorescence) as it evolves over time during the full experiment; long- and short-term pulse disturbances are indicated on the right side of the plot. Download MOVIE S1, MOV file, 8.8 MB. Copyright © 2018 Liu et al. 2018 Liu et al. This content is distributed under the terms of the Creative Commons Attribution 4.0 International license.

### Classifying ecological situations.

Grimm and Wissel (18) point to the importance of the ecological situation in which statements regarding stability are formulated. This ecological context can be described by six features, which we first need to define for our workflow in order to assess microbial community stability.

### (i) Level of description.

The level of description in our case refers to the whole microbial community, as flow cytometric data characterize the whole community by providing relative abundance information for gate-allocated subpopulations. This defines the structure of the community.

### (ii) Variable of interest.

In our case, the variable of interest is the relative community composition, which defines the structure of our microbial system at any given time. This structure (*s*) is the system state and can be defined as follows. Assume that for a given experiment, a gate template with *n* gates is appropriate to flow cytometrically characterize community dynamics over time. In this case, we get information on the relative abundance of each gate population at each sampling time. More generally, we then can define the state of our system at time *t* as

$$s(t):=\left[s^{i}\left(t\right),\cdots ,s^{n}\left(t\right)\right]$$

where *s*^*i*^(*t*) indicates the relative abundance of gate population *i* at time *t*. Due to dealing with relative abundances, we have

$$\sum_{i=1}^{n}s^{i}(t)=1$$

for all times *t*. Next, we need a way to quantify the difference between two community structures (*s_a_* and *s_b_*) (Text S3). It is a common approach to interpret such structures as points in *n*-dimensional space, so that the Euclidean distance (*d*_*e*_) provides a natural way of characterizing the deviation between two states. We additionally introduce a scaling factor $\left(1/\sqrt{2}\right)$ to ensure that deviations can be expressed as a value between 0 and 1:

$$d_{e}(s_{a},s_{b}):=\frac{1}{\sqrt{2}}\cdot \sqrt{\overset{n}{\underset{i=1}{\sum }}{\left(s_{b}^{i}-s_{a}^{i}\right)}^{2}}$$

where 0 ≤ *d*_*e*_ ≤ 1. While being straightforward to compute, this way of calculating the difference between states can be misleading, as it considers only absolute differences in gate populations. Consider a gate population with 100,000 cells. An addition or removal of 1,000 cells would constitute only a minor change for this population. However, if the gate population consists of 1,000 cells only, the removal or addition of 1,000 cells leads to the extinction or the doubling of this population, qualitatively a big change in the community structure. As the Euclidean distance cannot distinguish between these two cases, we propose the normalized Canberra distance (*d*_*c*_) (41) as an alternative way to compare structures (and later compare both approaches), as follows:

$$d_{c}(s_{a},s_{b}):=\frac{1}{n}\overset{n}{\underset{i=1}{\sum }}\frac{\left|s_{a}^{i}-s_{b}^{i}\right|}{s_{a}^{i}+s_{b}^{i}}$$

where 0 ≤ *d*_*c*_ ≤ 1. Here, the difference in gate populations is evaluated relative to their summed abundances, providing a more meaningful characterization of structure deviations.

10.1128/mSphere.00564-17.3 TEXT S3 Calculations of gate-based deviations in community structures over time. Download TEXT S3, PDF file, 0.3 MB. Copyright © 2018 Liu et al. 2018 Liu et al. This content is distributed under the terms of the Creative Commons Attribution 4.0 International license.

### (iii) Reference state or reference dynamic.

Ideally, more than one sample is available to characterize the system’s state prior to the disturbance. Such *m* reference samples, *s^r^*_1_, …, *s^r^_m_*, can be obtained as a time series before the disturbance event or from technical replicates. In this case, the reference state can be determined by taking the mean values of all gate populations from these samples (*s^r^*_1_, …, *s^r^_m_*) as follows:

$$s_{\text{ref}}:=\left(\bar{s^{r,1}},\cdots ,\bar{s^{r,n}}\right)$$

where $\overset{¯}{s^{r,i}}$ is the mean of gate population *i* over the population of all reference samples, *s^r^*_1_, …, *s^r^_m_*. As suggested by Pimm (42), the variability of the system can be assessed by determining the standard deviation. The standard deviations σ_1_, …, σ_*n*_ for all *n* gate populations over the reference samples provide a measure of the variability of the system in its predisturbance phase, defined as σ_ref_ := (σ_1_, …, σ_*n*_), which includes both the natural variability and the technical variability of the measurement process.

An *n*-dimensional sphere centered at *s*_ref_ can serve as a boundary whose crossing indicates the deviation of the system from its reference state. Likewise, a return to the reference state can be detected by a second crossing in the opposite direction. The radius (*r*) of this sphere is given by the maximal deviation from the reference state calculated over all reference state samples:

$$r_{e/c}:=\underset{s\in \{s_{1}^{r},\cdots ,s_{m}^{r}\}}{\max}[d_{e/c}(s_{\text{ref}},s)]$$

either using the Euclidean or the Canberra distance. The reference state taken together with this threshold defines the reference space, which characterizes the community’s predisturbance behavior. If only one sample prior to the disturbance event is available, this sample can alternatively serve as the reference state. In this case, a threshold must be selected based on previous experience.

With the reference state defined, the dynamics of the system can be followed by considering its deviation from this reference state over time *t*, calculated by the Euclidean distance *d_e_*, as follows:

$$d\left(t\right):=d_{e}\left[s\left(t\right),s_{\text{ref}}\right]$$

or by the Canberra distance (*d_c_*) likewise. Furthermore, we define the maximal deviation in response to a disturbance event by *d*_max_ = max[*d*(*t*)] and identify the time at which this maximal deviation occurs as *t*_max_ and the corresponding state as *s*_max_. The state at the end of the experiment is denoted as *s*_end_, with *t*_end_ and *d*_end_ defined likewise (Table 2).

**TABLE 2:**
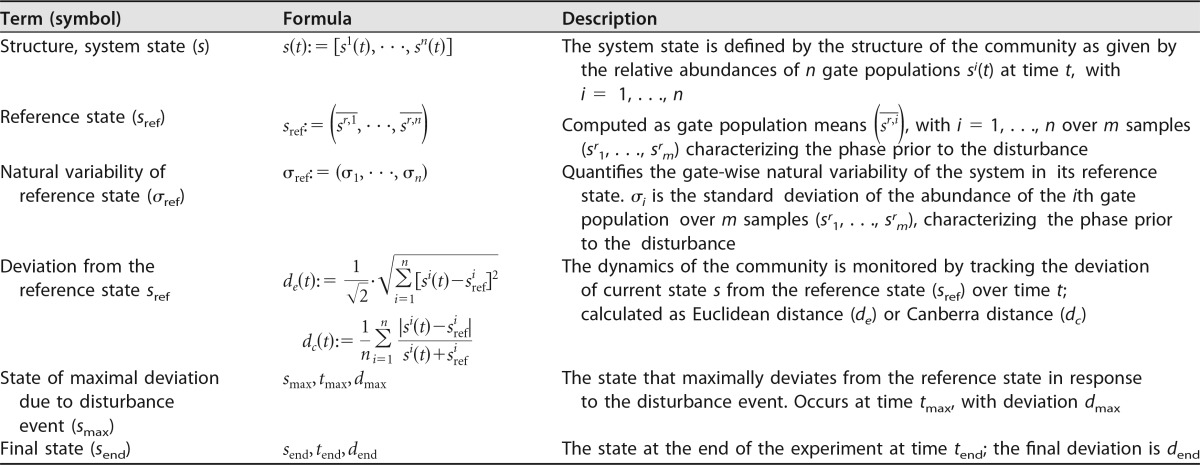
Formal definitions for characterizing NMC composition, reference state, and dynamics

### (iv) Disturbance, spatial scale, temporal scale.

The remaining three features, disturbance, spatial scale, and temporal scale, cannot be universally defined but depend on the respective experimental settings. For our continuous reactor experiment, the settings are described in Results and in Text S1. Table 2 summarizes the concepts introduced so far.

### Classifying stability properties.

**FIG 3:**
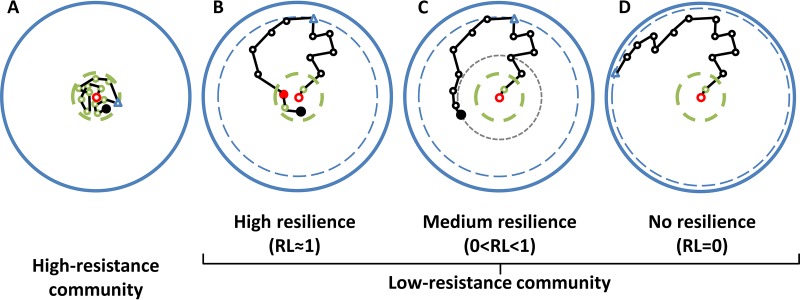
Potential community responses to a disturbance. The reference state characterizing the phase prior to the disturbance (*s*_ref_) is marked by the open red circle. Natural variability in the reference state (σ_ref_) is indicated by a dashed green line. Symbols connected by black lines indicate how community structure changes in response to the disturbance. The state maximally deviating from the reference state (*s*_max_) is marked by a blue triangle, with a corresponding blue dashed line at its radius (*d*_max_) showing the maximum deviation from* s*_*ref*_. The solid blue line marks the theoretical *s*_max_ value. The final structure, *s*_end_, is marked by a filled black circle. (A) For a microbial community of high resistance (RS values close to 1), the disturbance does not lead to a change in the community structure, and the system remains within the limits of its natural variability. (B and C) For systems of lower resistance, the disturbance leads to a change in community structure beyond its natural variability. For resilient systems (RL > 0), after passing through* s*_max_, the community structure either returns to the reference state, with the filled red circle indicating this return into the reference space (RL close to 1) (B), or at least approaches it so that *d*_end_ is <*d*_max_ (0 < RL < 1) (C). (D) If the system is not resilient at all (RL = 0), its state continues to deviate more from the reference state, with *d*_end_ equal to *d*_max_.

The next step in the workflow is considering systems that have the property of constancy and for which a suitable reference state is obtainable, that is, featuring a constant composition in the reference phase prior to the disturbance, as evidenced by low values for σ_ref_. Continuing in the workflow, we propose flow cytometric data-based quantification methods for four stability properties (Table 1). (i) Resistance (RS) refers to the ability of a system to remain mostly unchanged in the face of a disturbance. We quantify this property by the maximal deviation from the reference state caused by a disturbance. (ii) The displacement speed (DS) indicates how fast a system is displaced upon disturbance. This is given by the maximal distance divided by the time required for the shift from the reference state (*s*_ref_) to the maximally deviating state (*s*_max_). (iii) Resilience (RL) is the ability of the system to return to the reference state after a disturbance. For its quantification, we use the definition given by Orwin and Wardle (43). Resilience is computed as an index over time, with values ranging between −1 and 1. Given a disturbance-caused displacement, positive values indicate resilient behavior in which the system again approaches the reference state, with a value of 1 indicating full recovery. Negative values indicate nonresilient behavior where the system, after the initial displacement, continues to depart further from the reference state due to internal dynamics triggered by the disturbance. In our approach, we do not distinguish such events but attribute the maximally observed deviation (*d*_max_) from *s*_ref_ to the disturbances such that negative values cannot occur. For comparing different experiments, resilience can be computed as a single value for the final state of the system (*s*_end_). Besides introducing this *post hoc* analysis, we introduce a modified method to compute resilience which can be applied as an online monitoring tool (Text S4). For online resilience computation, we replace *d*_max_ in the resilience equation (Table 1) by the maximal distance that has been encountered in the experiment so far. Given that ongoing variations in community structure lead to increases beyond the previously encountered maximal distance, online resilience is evaluated to be zero. Only as a maximal deviation is reached and the system again approaches the reference state, positive online resilience values occur. Hence, a value of zero indicates that the system is continuing to deviate more from the reference state, while positive values indicate the onset of recovery, with a value of 1 indicating perfect recovery, as in *post hoc* analysis. (iv) Elasticity (E) indicates the speed of recovery after a disturbance-caused displacement. This is calculated as the distance traveled from *s*_max_ toward the reference state divided by the elapsed time. To clarify the proposed concepts, potential community dynamics featuring high and low levels of resistance in combination with various levels of resilience are schematically shown in Fig. 3. To easily apply the proposed quantification method, an R script is available for download at GitHub (https://github.com/fcentler/EcologicalStabilityPropertiesComputation/) (Text S4).

10.1128/mSphere.00564-17.4 TEXT S4 R script for calculation of stability properties. Download TEXT S4, PDF file, 0.3 MB. Copyright © 2018 Liu et al. 2018 Liu et al. This content is distributed under the terms of the Creative Commons Attribution 4.0 International license.

10.1128/mSphere.00564-17.5 TEXT S5 Sequence-based analysis. Download TEXT S5, PDF file, 1.1 MB. Copyright © 2018 Liu et al. 2018 Liu et al. This content is distributed under the terms of the Creative Commons Attribution 4.0 International license.

10.1128/mSphere.00564-17.6 TEXT S6 Diversity metrics of cytometric data. Download TEXT S6, PDF file, 0.04 MB. Copyright © 2018 Liu et al. 2018 Liu et al. This content is distributed under the terms of the Creative Commons Attribution 4.0 International license.

### Sequencing.

Sequencing is included in the workflow to provide controls based on 16S rRNA gene amplicon sequencing to support cytometric data. Information on samples analyzed by Illumina 16S rRNA gene amplicon sequencing is given in Text S5, section S5.1. From those samples, DNA was extracted and tested for quality (Text S5, section S5.2). The preparation of DNA from samples for Illumina sequencing is described in Text S5, section S5.3, while the evaluation and discussion of the data are presented in Text S5, section S5.4. In short, the CMC and gates thereof showed generally higher operational taxonomic unit (OUT) numbers than the AMC, as expected. The influence of cell fixation procedures on the quality of sequencing results is shown in Text 5, section S5.5.

### Data availability.

Cytometric data were uploaded into the Flow Repository database (https://flowrepository.org/) under accession number FR-FCM-ZZTV (http://flowrepository.org/id/RvFrs5hE0ALnokont7Z8GiOEZTIBr93jvwBFQeOcLxlDPSTRVvhdtB7ZiOJ1oi3p). Version 1.0 of the R script implementing the proposed concepts is available for download at GitHub (https://github.com/fcentler/EcologicalStabilityPropertiesComputation). The tool flowCybar is available on the Bioconductor platform, http://www.bioconductor.org/packages/release/bioc/html/flowCyBar.html. Sequencing data can be found in the NCBI database under BioProject accession number PRJNA407269.
