# Supplementary material for: Ecological Stability Properties of Microbial Communities Assessed by Flow Cytometry
Source: mSphere. 2018 Jan 17;3(1):e00564-17. doi: 10.1128/mSphere.00564-17 (PMC5770544; doi:10.1128/mSphere.00564-17)
Supplement: TEXT S1 [file sph001182449s1.pdf]

Ecological stability properties of microbial communities assessed by flow cytometry

Zishu Liu[1], Nicolas Cichocki[1], Fabian Bonk, Susanne Günther, Florian Schattenberg, Hauke Harms, Florian Centler, Susann Müller

UFZ – Helmholtz Centre for Environmental Research, Department of Environmental Microbiology, Permoserstr. 15, 04318 Leipzig, Germany.
[1]divided first authorship

Supplemental Text S1

S1: Bacterial strains and culture conditions

The mock strains 1 and 2 correspond to *Rhodococcus sp*. RAH1 and *Pseudomonas putida* KT2440, respectively, and were used as positive controls for the MiSeq sequencing run. The SILVA data base version 123 (Quast et al. 2013, Text S5 in the supplemental material, Section S5.4) recognized them on the genus level as *Rhodococcus* and *Pseudomonas*. *Rhodococcus sp*. RAH1 was obtained from the strain collection of the Helmholtz Centre for Environmental Research (Leipzig, Germany) while *P. putida* KT2440 was obtained from the German Collection of Microorganisms and Cell Cultures (Braunschweig, Germany). The strains were cultivated aerobically in 500-mL flasks on a rotary shaker at 30 °C and 150 rpm in separate batches of 100 mL Lysogeny broth: tryptone 10 g L^{-1} (Oxoid, Hampshire, United Kingdom), yeast extract 5 g L^{-1} (Difco, Detroit, Michigan, USA), and NaCl 10 g L^{-1}.

The three members of the AMC were also classified using the SILVA data base version 123 and affiliated to either with the genera *Bacillus* and *Paenibacillus*, or family level as *Comamonadaceae*. The AMC was pre-cultivated in 500-mL shake flasks (150 rpm) with 150 mL in Lysogeny broth (see above) at 30 °C and pH 7. For the CMC an activated sludge basin was sampled at the communal wastewater treatment plant (WWTP) in Eilenburg, Germany (51°27'39.4"N, 12°36'17.5"E, ~10200 m^3 wastewater per day, water purification according to German law (Waste Water Ordinance – AbwV 2004)). The wastewater was frozen into aliquots at -20°C and one of the aliquots (10 mL) was pre-cultured (150 rpm, overnight, at 30 °C) in 100ml 2 % peptone and 98 % synthetic wastewater medium: 0.198 g L^{-1} peptone, 0.2 g L^{-1} meat

extract, 0.219 g L^{-1} yeast extract, 0.1 g L^{-1} glucose, 0.49 g L^{-1} Na-propionate, 0.0059 g L^{-1} $CaCl_2 2H_2O$, 0.0294 g L^{-1} KCl, 0.06 g L^{-1} NaCl, 0.04 g L^{-1} K_2HPO_4, 0.2156 g L^{-1} KH_2PO_4 and 0.0196 g L^{-1} $MgSO_4 7H_2O$. This pre-culture was used as CMC. The phylogenetic affiliation of their members are listed in Table S5.2 and Figure S5.1 in Text S5 in the supplemental material.

Continuous reactor cultivation of AMC and CMC

Continuous reactor cultivations were carried out in a Biostat MD laboratory stirring bioreactor (BIOSTAT® B-DCU II, Sartorius, Goettingen, Germany) with a final working volume of 1 L. Environmental conditions were set to 30 °C, a stirrer speed of 400 rpm, an air pressure of 1.2 bar, and an aeration rate of 2.0 L min^{-1}. Dilution rate was adjusted to 0.036 h^{-1} (0.6 mL min^{-1} Lysogeny broth), and controlled during the whole experiment by a peristaltic pump (Watson Marlow, 101U/R, Falmouth, England) and a volume controller inside the reactor. The pH was kept constant at pH 7.0 by adding 1M KOH or 1M H_2SO_4 as required and measured online in the liquid phase (Ingold, Mettler Toledo GmbH, Giessen, Germany).

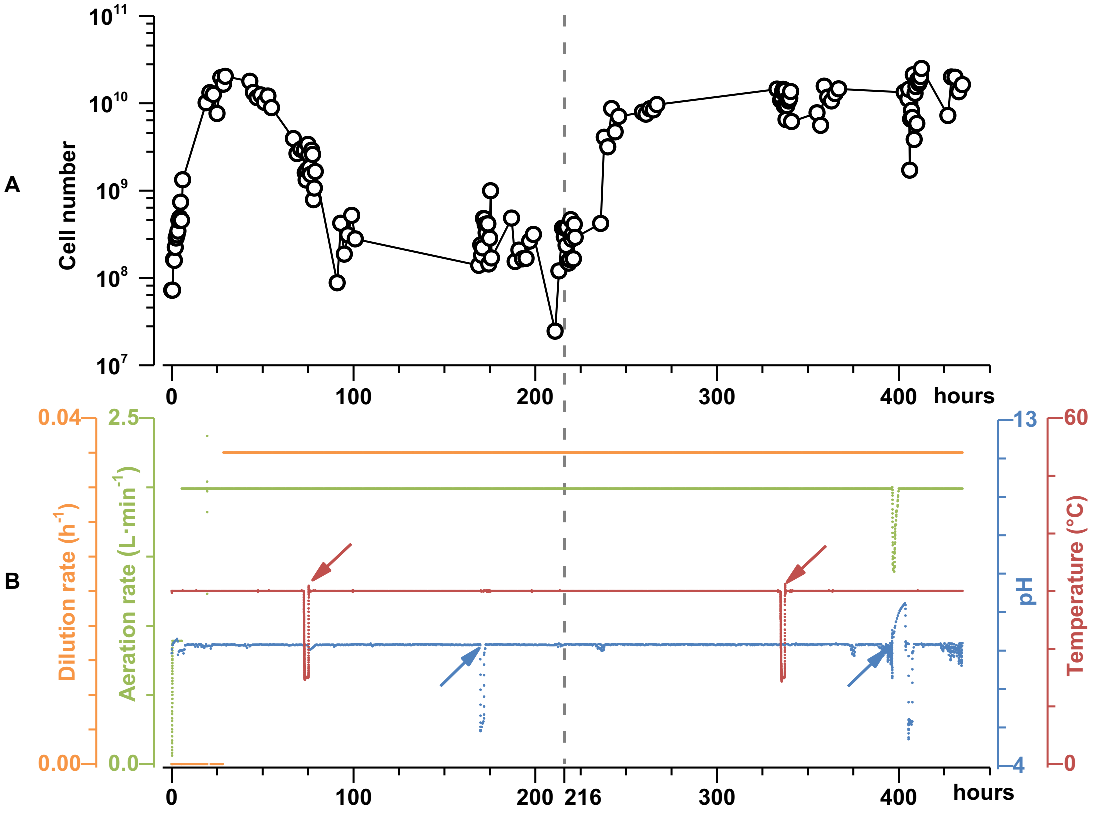

Figure S1.1: Design of disturbace experiment. **(A)**: Cell numbers were counted with identical workflow for AMC and CMC samples (Text S2 in the supplemental material, Section S2.1). **(B)**: Dilution rate, aeration rate, pH, and temperature. Blue arrows indicate pH disturbance while red arrows indicate temperature disturbance. The grey

vertical line indicates the shift from the artificial microbial community (AMC) to the complex microbial community (CMC).

An artificial microbial community (AMC, constructed by three strains detected on the genus level as *Bacillus* and *Paenibacillus*, as well on the family level as *Comamonadaceae* on the SILVA data base version 123, Quast et al. 2013, see Text S5 in the supplemental material, Sections S5.1 and S5.4), was cultivated in a continuous bioreactor for 216 h. A long-term disturbance was simulated by a single invasion event (addition of 60 ml wastewater pre-culture) of a complex microbial community (CMC) which resided for another 219 h. In addition, short term pulse disturbances (2 h, respectively) were simulated by temperature changes from 30 °C to 15 °C (73 - 75 h and 335 - 337 h) and for pH from pH 7 to pH 5 (170 - 172 h). A second pH disturbance was slightly raised first from pH 7 to pH 8.2 (396 - 404 h) and then this value was reduced to pH 5 similar to the other disturbance before (404 - 407 h). With exception of the pulse disturbances periods the pH and the temperature were kept constant. Cell number was increasing during the first 29.5 h from 7.23×10^7 to 2.04×10^{10} cells mL^{-1} but decreased constantly until reaching a plateau of about 3×10^8 cells mL^{-1} for the AMC (91 h to 215 h). The addition of the CMC led to a stark increase in cell numbers up to average of 1.14×10^{10} cells mL^{-1} (for 238 h to 435 h) after an adaptation period of 22 hours.

References

Quast, C., Pruesse, E., Yilmaz, P., Gerken, J., Schweer, T., Yarza, P. , Peplies, J. & Glöckner, F.O. (2013) The SILVA ribosomal RNA gene database project: improved data processing and web-based tools. *Nucleic Acids Research,* 41, 590-596.

Waste Water Ordinance - AbwV (2004), Federal Republic of Germany, Apendix 1, Municipalities / domestic wastewater
