# Supplementary material for: Ecological Stability Properties of Microbial Communities Assessed by Flow Cytometry
Source: mSphere. 2018 Jan 17;3(1):e00564-17. doi: 10.1128/mSphere.00564-17 (PMC5770544; doi:10.1128/mSphere.00564-17)
Supplement: TEXT S2 [file sph001182449s2.pdf]

Ecological stability properties of microbial communities assessed by flow cytometry

Zishu Liu[1], Nicolas Cichocki[1], Fabian Bonk, Susanne Günther, Florian Schattenberg, Hauke Harms, Florian Centler, Susann Müller

UFZ – Helmholtz Centre for Environmental Research, Department of Environmental Microbiology, Permoserstr. 15, 04318 Leipzig, Germany.
[1]divided first authorship

Supplemental Text S2

S2: Flow cytometric analysis

S2.1: Harvesting, fixation, and staining procedure for flow cytometry

Harvesting:
Samples were harvested from the bioreactor via a specific silicone membrane port under sterile conditions by using sterile needles and syringes. At each sampling time two samples of 2.2 mL were taken for the biological replication procedure. This harvesting procedure was done every 30 min, during the first 6 h after each disturbance (pH, temperature) and every 2 h otherwise (excluding nights and weekends).

Fixation:
Samples were centrifuged (3 200 g, 10 min, 4 °C) and the supernatant was discarded. The cells were washed in phosphate buffered saline (PBS: 6 mM Na_2HPO_4, 1.8 mM NaH_2PO_4, 145 mM NaCl, pH 7) once (3 200 g, 10 min, 4 °C) and stabilized by adding a para-formaldehyde solution (PFA, 2 % in PBS) to the cell pellet and incubated for 30 min at room temperature (RT). After another centrifugation step (3 200 g, 10 min, 4 °C), 2 mL of ethanol (70 %) were added for fixation and the cell solution stored at -20 °C for two months maximum.

Staining:

For the staining procedure, the cells were taken out of the fixation solution (70 % ethanol), centrifuged, washed in PBS, centrifuged again, and the optical density (OD) of the cells adjusted to 0.035 ($d_{\lambda 700nm}$ = 0.5 cm) in 2 mL PBS (6 mM Na_2HPO_4, 1.8 mM NaH_2PO_4, 145 mM NaCl, pH 7). Then, the samples were washed another time (3 200 g, 10 min, 4 °C in PBS) and 1 mL of solution A (citric acid 0.11 M, Tween 20, 4.1 mM, in bidistilled water) was added. Incubation took place for 20 min at RT; 10 min in a ultrasonication water bath for CMC samples (ultrasonic bath, Merck Eurolab, Darmstadt, Germany, at RT), and 10 min on the lab bench for AMC samples. After another centrifugation step (3 200 g, 10 min, 4°C) the cells were stained with 0.24 µM DAPI (4',6-di-amidino-2-phenyl-indole, Sigma-Aldrich, St. Louis, USA) in 417 mM Na_2HPO_4/NaH_2PO_4 buffer (289 mM Na_2HPO_4, 128 mM NaH_2PO_4, pH 7) overnight in the dark at RT. The stained samples were filtered using 50 µm CellTrics filter (Sysmex Partec GmbH, Görlitz, Germany) before measurement to prevent clogging of the cytometer nozzle (70 µm).

The influence of the sonication procedure on the cytometric community structure was tested for one sample (Fig. S2.1). One untreated sample (A) was compared to one subjected to sonic treatment (B) and sonic treatment during incubation in solution A (C). As shown below the differences between the three approaches were only small, and we decided to use treatment C.

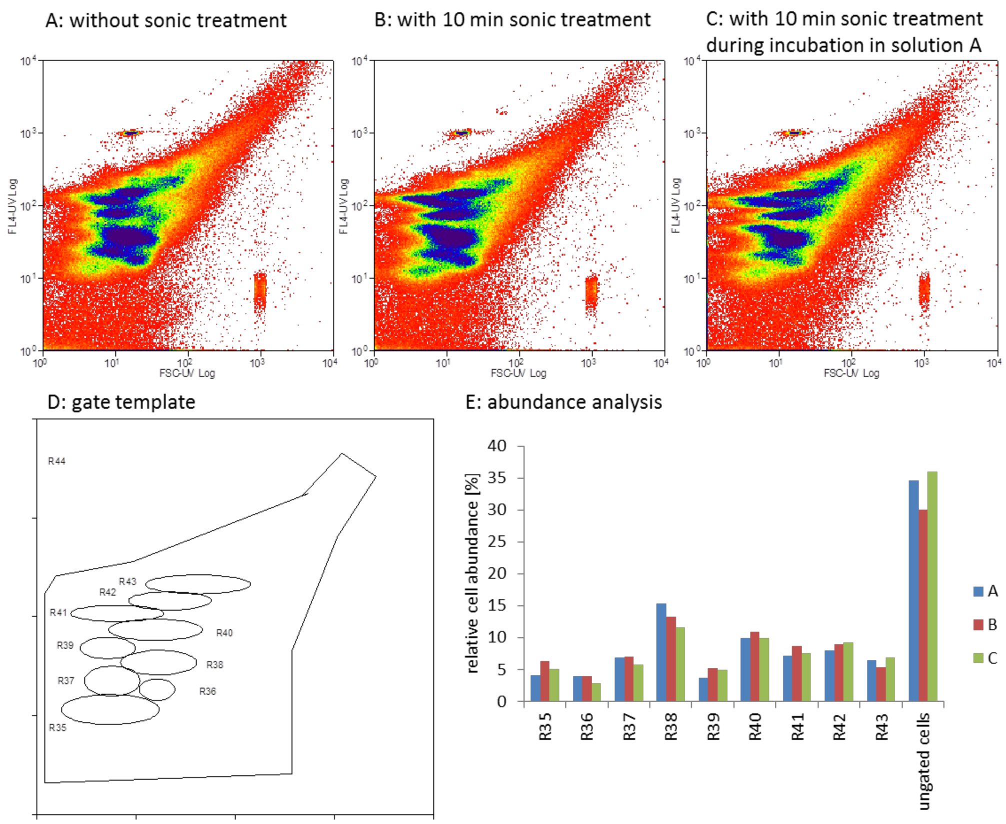

Figure S2.1: Testing of sonication on cytometric community patterns from a cultivated pre-test CMC: **(A)** untreated sample, **(B)** treatment with 10 min sonication before OD adjustment, **(C)** treatment with 10 min sonication during incubation in solution A. **(D)** gate template for abundance analysis, **(E)** relative abundance of cells in gates.

Cell Analysis:

Samples were measured with a MoFlo Legacy cell sorter (Beckman-Coulter, Brea, California, USA) which is equipped with two lasers. The 488nm laser Genesis MX488-500 STM OPS (Coherent, Santa Clara, California, USA) at 400 mW was used for measurement of FSC (bandpass filter 488/10, neutral density filter 1.9) and SSC signal (bandpass filter 488/10, neutral density filter 1.9, trigger signal) and the 355nm UV laser Xcyte CY-355-150 (Lumentum, Milpitas, California, USA) at 150 mW for UV-induced fluorescence (bandpass filter 450/65). Photomultiplier tubes were purchased from Hamamatsu Photonics (Models R928 and R3896; Hamamatsu City, Japan). Daily and in-between-day calibration of the instrument was performed with fluorescent 1 µm UV beads (FluoSpheres (350/440)) and 2 µm yellow-green beads (FluoSpheres (505/515), both from Molecular Probes (Eugene, Oregon, USA). UV beads (0.5 µm and 1 µm, both Fluoresbrite BB Carboxylate microspheres, (360/407), PolyScience, Niles, Illinois, USA) were added to each sample.

DNA-stained samples were measured flow cytometrically as logarithmically scaled 2D-dot plots according to DAPI fluorescence for DNA content and forward scatter for cell size related information. For every 2D-dot plot 250 000 cells were measured (see Section S2.4) and beads (0.5 µm and 1 µm UV beads, Fluoresbrite BB Carboxylate microspheres (360/407), PolyScience, Niles, USA) were amended into the sample for adjustment. Cell numbers of the cell suspensions were determined using a defined number of 1 µm yellow-green beads (YG beads, FluoSpheres (505/515), Molecular Probes, Eugene, USA) measured together with a defined volume of DNA-stained cells. The 2D-dot plots were gated with regard to cells and beads (gating strategy see Section S2.4), and cell counts were calculated as follows:

$$\text{Cell number mL}^{-1} = \frac{f \cdot C(\text{parent}) \cdot B \cdot V}{B(\text{YG}) \cdot V(\text{sample})}$$

f: The dilution rate of sample for counting

C(parent): The virtual cell number in the parent gate

B: The defined concentration of 1 µm YG beads

V: The volume of defined concentration 1 µm YG beads

B(YG): The number of beads in the gate 1 µm YG beads

V(sample): The defined volume of DNA- stained cells sample

Cell Sorting:

The cell sorting procedure was done as follows: 70 % ethanol fixed samples were washed in PBS and stained according to the procedure described above. After setting gates for sorting, the sort procedure was started. Each sorted sample was composed of 500 000 cells for every gate selected for sorting. The sorting procedure was done in the most accurate sort mode of the MoFlo (highest-purity sort mode single-cell and one-drop: purity 99 %) at a rate not higher than 2 500 particles per second. Cells were harvested by a centrifugation step (20 000 g, 4 °C, 25 min), and the pellet was frozen at -20 °C for later pooled DNA extraction, library preparation and MiSeq sequencing.

S2.2: Cytometric terms used in the study

Cytometric terms used in this study are collected in Box S2.1. Some of the terms were newly defined while others were described in an earlier work (Koch et al. 2014).

Box S2.1: Cytometric terms

FSC: Forward scatter is an optical characteristic containing information related to cell size.
SSC: Side scatter is an optical characteristic containing information related to cell density.
DAPI fluorescence: Is an optical characteristic that is used for quantification of cellular DNA content.
Event: Can be a cell, a bead, or noise in a cytometric histogram.
Beads: Monodispersed microspheres are used for calibration of the instrument, the alignment of the 2D-dot plots, and for cell number determination.
Cell: The microbial cell is an individual biological unit. It is characterized by optical characteristics which can be measured using flow cytometry.
Virtual cell: The virtual cell represents the cell's characteristics regarding the chosen optical parameters usually in a 2D-dot plot.
Community: Is the entity of microorganisms in a natural sample. It can comprise high diversity, i.e., hundreds of different species regarding phylogeny and function.
Subcommunity/Cluster: Virtual cells with similar optical properties.
Gate: A gate marks a cluster of cells in the histogram that differ from others in their optical properties. It can be defined using one, two, or even more parameters.
Gate-template: Represents the entity of all gates. It is defined by marking all upcoming clusters of one defined experimental series and finally applied to all samples within this experiment.
Cell sorting: Separation of selected cells out of a community using a cell sorter. Cells were further processed by Illumina sequencing in this study.

S2.3: Intrinsic variation of technical samples in flow cytometric patterns

To verify the reliability of cytometric measurements a sample is splitted into three parts and treated independently according to the cytometric workflow. As an outcome three parallel 2D-dot plots are produced that are evaluated by using the gate-template (see Section S2.4). Numbers of cells per gate are calculated by using the program FlowJo (FlowJo LLC, Oregon, USA). The relative abundance of a gate (i.e. cells belonging to this gate given as a fraction of the total population, expressed in %) varied between the three technical replicates, with a mean standard deviation over all gates of 0.6 %, and a maximal standard deviation of 4.5 %.

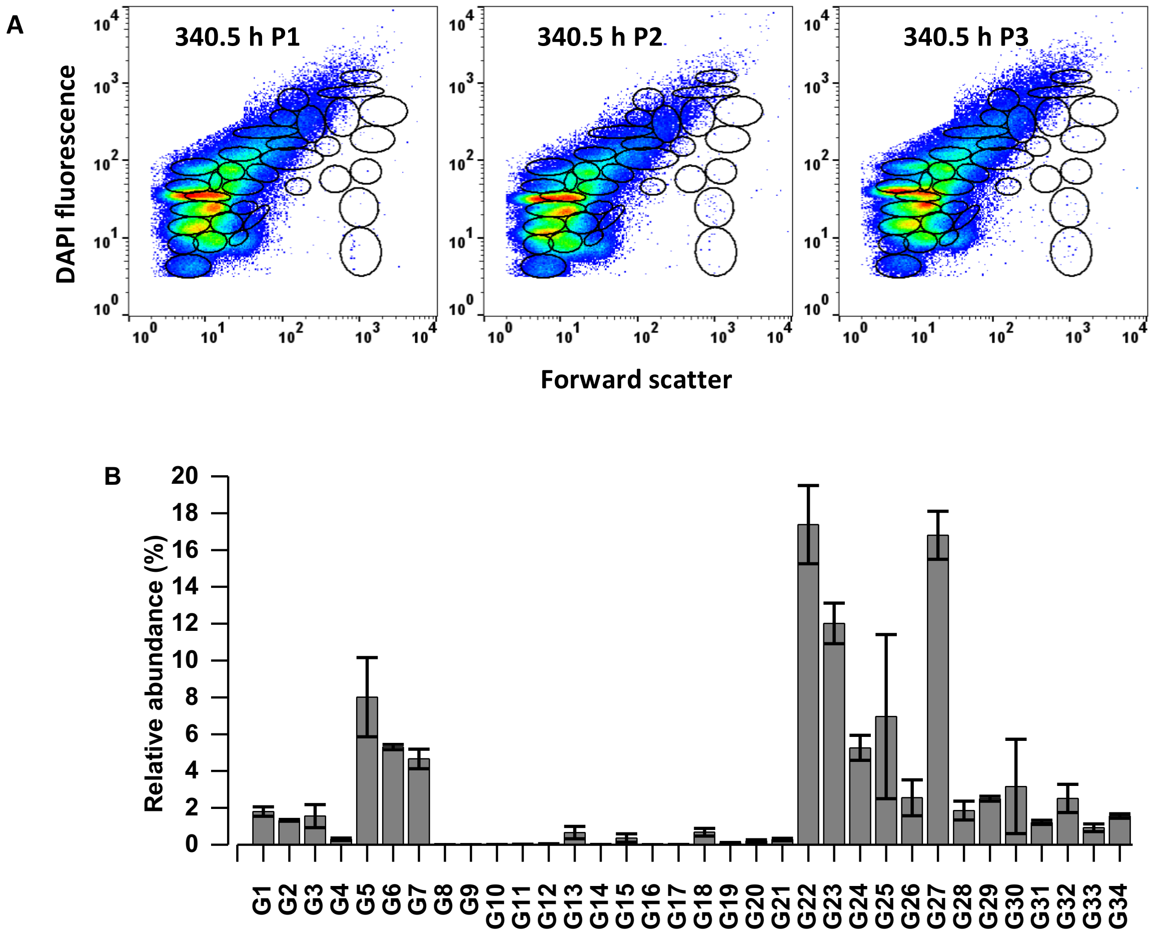

Figure S2.2: (A) Exemplary cytometric 2D-dot plots of three DAPI stained CMC samples (P1 - P3) which were harvested at 340.5 h. The gate-template (see Section S2.4) is shown as overlay in the 2D-dot plots. **(B)** Variation analysis of flow cytometric measurement. One sample was measured thrice and the average relative abundance as well as standard deviation calculated for each gate.

S2.4: Gate-template for evaluation of AMC and CMC distributions.

Gates are set according to virtual cell clusters for all measurements within the continuous reactor experiment.

Relative abundancies were determined by setting the sum of all gates as 100 %.

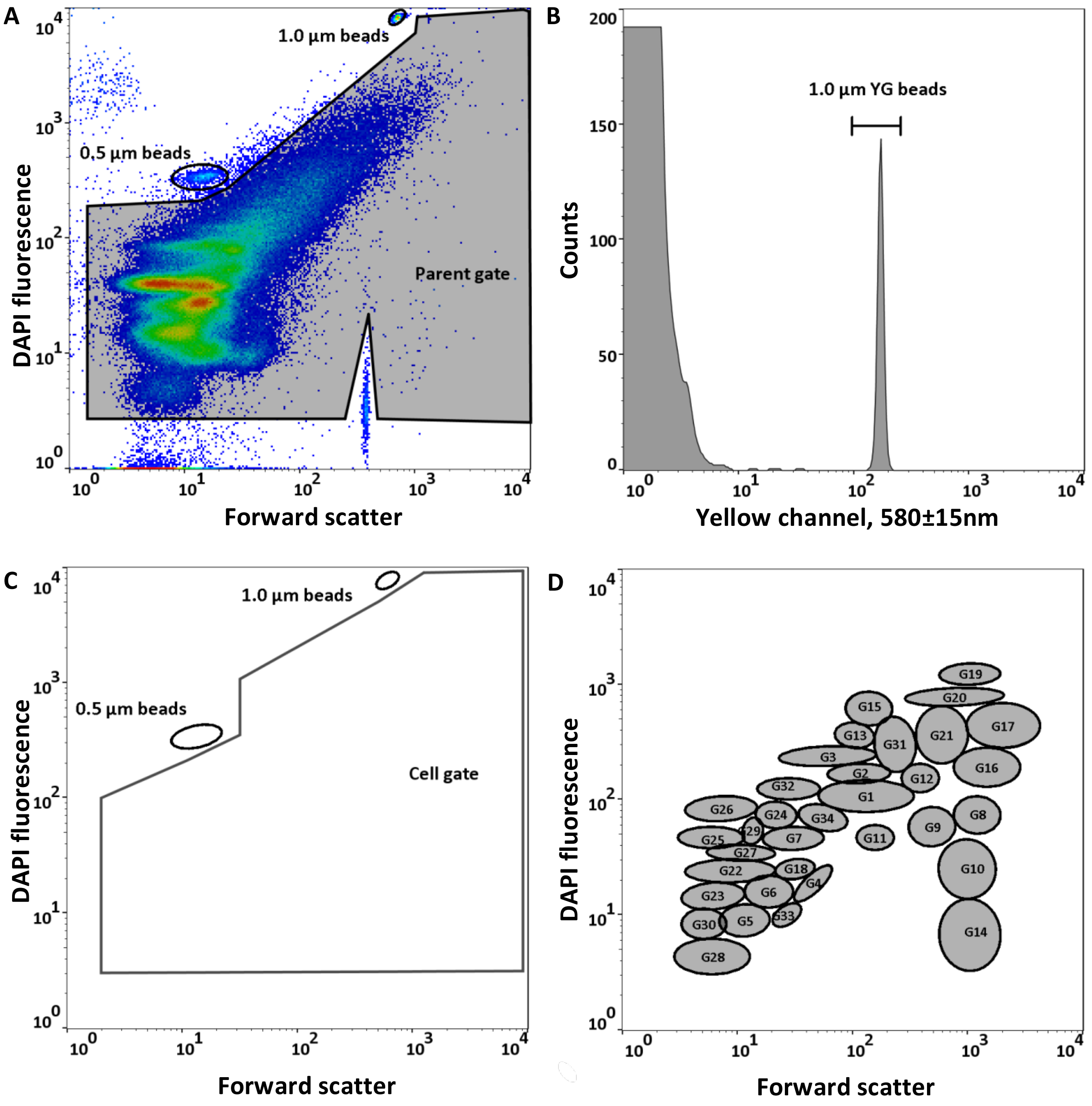

Figure S2.3: (A) 250 000 cells were measured for each sample using a parent gate created in Summit Ver. 4.3 (Beckman-Coulter, Brea, CA) which comprises all stained cells and excludes noise and beads. The example shows the community at hour 340.5 of the experiment. **(B)** For total cell number estimation 1 µm yellow green (YG) beads were amended into each sample and the bead number measured by using gate "1.0 µm YG beads" in the yellow channel of the flow cytometer (band pass filter 580 ± 15 nm). Total cell counts were determined as described in Section S2.1. **(C)** For sample analysis the software in FlowJo V10 (FlowJo LLC, Oregon, USA) was used. Therein a cell gate similar to the parent gate used in A was created to remove virtually noise and beads from the original 2D-dot plots. **(D)** All 140 samples together were used to create the gate-template for the community which contained between about 200 000 and 250 000 cells of the parent gate. A gate was set wherever a new subcommunity

became apparent. The final gate-template was then applied to each sample to extract individual subcommunity abundances.

S2.5: Tables for individual amounts of cell numbers in gates

Tables S2.1 to S2.10 show abundancies of cells per gate for all 140 cytometrically measured samples.

Table S2.1: Absolute cell numbers in gates from G1 to G34 of samples picked between 0 and 19 h

Time	0 h	0.5 h	1 h	1.5 h	2 h	2.5 h	3 h	3.5 h	4 h	4.5 h	5 h	5.5 h	6 h	19 h
**G1**	2571	3827	5004	2340	3931	2306	2041	1956	1651	1262	2849	1488	2220	1043
**G2**	464	798	867	544	900	506	539	799	1042	1004	2362	1572	2450	330
**G3**	1130	1837	2305	1419	2006	2023	2149	2492	3283	2480	4220	2679	3578	444
**G4**	112035	135893	132204	152078	140496	154234	153787	142179	140715	137985	128920	137934	134493	3685
**G5**	14469	5752	4980	2250	3430	2171	1440	2102	2462	2007	3583	2300	3057	135353
**G6**	34335	15765	23906	19275	23319	18273	16012	14987	17515	12681	28351	21436	21832	75195
**G7**	329	306	666	533	573	1110	2675	1596	3224	2399	838	2275	1635	202
**G8**	27	10	6	8	8	7	7	3	2	3	3	13	3	0
**G9**	35	30	30	23	16	26	23	9	17	8	9	16	8	1
**G10**	70	46	19	19	25	13	18	19	11	14	18	26	10	5
**G11**	84	118	311	319	173	311	366	245	295	276	258	266	257	20
**G12**	65	61	54	68	26	23	40	23	37	34	12	24	20	15
**G13**	115	147	218	222	291	852	883	1256	1455	1039	966	742	988	48
**G14**	94	30	30	44	34	28	28	30	31	23	28	36	34	10
**G15**	55	60	84	91	45	181	316	286	359	320	285	291	396	24
**G16**	29	18	17	10	13	9	11	11	8	17	3	9	17	1
**G17**	37	35	18	35	21	6	19	9	16	24	4	7	5	2
**G18**	10053	3592	11300	8057	8994	11676	12456	6647	10133	6659	12442	9844	9714	4522
**G19**	41	48	78	154	44	51	75	57	80	76	37	68	121	16
**G20**	73	105	127	225	34	78	137	64	120	97	44	121	170	19
**G21**	198	276	261	263	60	115	162	69	110	117	65	128	101	47
**G22**	246	208	800	311	676	475	317	509	356	263	625	372	436	671
**G23**	241	138	456	219	284	280	215	329	320	117	297	168	128	1314
**G24**	114	131	381	148	330	228	178	208	176	123	260	187	219	20
**G25**	18	11	72	44	46	31	32	38	45	14	27	22	17	41
**G26**	16	19	67	23	26	43	43	43	16	15	25	19	8	50
**G27**	111	89	448	187	370	288	195	276	255	157	334	254	250	77
**G28**	559	394	905	591	717	687	444	746	512	251	503	369	347	507
**G29**	34	30	168	46	121	82	57	94	82	48	83	88	93	11
**G30**	727	235	513	301	315	321	236	366	380	190	260	205	133	16898
**G31**	449	559	615	603	278	316	376	315	417	348	341	508	548	128
**G32**	167	302	588	238	566	430	302	297	234	133	370	103	103	118
**G33**	50182	53168	41983	34048	37327	27576	21833	30600	25003	31097	32264	31421	33944	5487
**G34**	158	145	249	173	301	234	217	211	279	201	114	287	205	47
**Sum**	229331	224183	229730	224909	225796	224990	217629	208871	210641	201482	220800	215278	217540	246351

Table S2.2: Absolute cell numbers in gates from G1 to G34 of samples picked between 21 and 67 h

Time	21 h	23 h	25 h	27 h	28.5 h	29.5 h	43 h	45 h	47 h	49 h	51 h	53 h	55 h	67 h
**G1**	852	666	444	581	753	761	1349	972	380	1652	344	413	565	4965
**G2**	255	139	127	119	177	287	119	81	69	127	56	57	56	1044
**G3**	293	153	212	154	123	99	46	19	19	27	19	16	17	386
**G4**	2675	1140	552	601	380	662	982	1533	1735	675	1084	805	354	2770
**G5**	130329	131491	139140	151729	150304	152238	119730	106004	111081	117208	107493	101046	90556	41523
**G6**	81009	79450	61940	54528	58587	53036	88388	70235	87305	79272	96936	103064	106370	50991
**G7**	125	240	211	103	246	154	118	66	105	113	132	128	88	4305
**G8**	0	1	1	0	3	3	1	0	0	12	0	0	8	109
**G9**	1	0	3	4	0	3	12	11	1	41	2	1	9	72
**G10**	7	1	1	6	2	8	8	2	6	1	6	6	11	59
**G11**	15	30	41	38	37	31	39	69	60	63	36	54	48	236
**G12**	15	31	31	32	43	25	45	60	53	103	39	41	53	316
**G13**	54	33	30	13	15	12	15	7	1	6	5	4	4	124
**G14**	11	16	14	18	10	7	7	14	13	16	14	9	8	154
**G15**	16	18	8	10	2	1	1	2	2	0	1	1	3	29
**G16**	1	0	2	3	4	2	1	8	2	12	1	1	4	94
**G17**	3	1	4	4	6	4	1	16	6	12	5	7	16	103
**G18**	2919	3917	3390	1787	2646	1501	2314	454	917	735	1114	1002	887	2846
**G19**	15	18	7	7	4	2	3	5	13	4	11	5	8	31
**G20**	21	28	16	19	16	5	7	16	30	10	25	19	16	116
**G21**	57	79	43	58	41	35	41	93	87	115	56	62	76	582
**G22**	849	1879	2727	1364	2775	1245	2056	313	528	538	854	790	1456	14214
**G23**	1249	2675	4612	2675	4523	2496	5269	1299	1847	2628	2770	3536	7432	38318
**G24**	14	28	21	18	34	26	9	3	17	8	5	9	10	1024
**G25**	18	20	17	13	17	16	2	4	3	6	9	3	3	333
**G26**	15	13	15	3	8	10	2	2	1	1	1	1	2	121
**G27**	92	216	151	112	244	117	145	33	72	57	96	88	98	3052

**G28**	884	612	1170	1017	359	1311	1260	13899	6952	5881	5025	5776	5233	6783
**G29**	8	11	12	12	11	19	9	6	3	5	4	6	7	494
**G30**	21292	29733	43329	40639	35624	36960	32632	20378	23216	34774	25747	24636	33130	61240
**G31**	133	135	88	97	80	56	48	67	63	85	71	66	63	859
**G32**	89	27	16	15	26	42	18	5	4	14	5	8	10	545
**G33**	4502	2127	924	1716	1691	2420	762	6732	3167	1571	1711	1695	1114	1404
**G34**	36	70	67	62	104	88	62	29	74	70	63	76	51	2193
**Sum**	247854	254998	259366	257557	258895	253682	255501	222437	237832	245842	243740	243431	247766	241435

Table S2.3: Absolute cell numbers in gates from G1 to G34 of samples picked between 69 and 78.5 h

Time	69 h	71 h	73 h	73.5 h	74 h	74.5 h	75 h	75.5 h	76 h	76.5 h	77 h	77.5 h	78 h	78.5 h
**G1**	1900	8843	4124	1705	1120	4306	5372	1304	3039	3012	5247	3975	1846	5560
**G2**	167	624	629	244	223	548	1331	650	1513	1972	3355	2350	653	1415
**G3**	50	161	224	89	86	203	310	157	191	427	2108	784	308	506
**G4**	5216	2363	2067	3304	3616	3600	1716	2166	2149	1667	1174	1365	1724	1265
**G5**	58489	42220	57780	63621	67200	59536	53317	59772	61477	56822	62172	59160	67387	61107
**G6**	87901	56481	67254	88343	95897	84726	55121	96438	76951	63565	68443	68052	83645	64428
**G7**	596	2092	1849	1009	1199	999	2686	939	684	1527	1881	1337	1467	1947
**G8**	101	6021	475	40	14	1172	325	14	589	1476	1226	58	52	806
**G9**	113	4607	236	27	18	661	164	12	431	874	602	31	18	389
**G10**	268	1353	222	411	432	875	296	726	625	786	1085	692	868	747
**G11**	91	1382	91	48	57	238	107	16	57	80	69	92	67	101
**G12**	129	886	283	69	80	231	587	42	149	241	509	269	144	317
**G13**	12	45	54	37	48	62	69	77	64	119	206	177	138	155
**G14**	25	195	258	81	110	63	118	139	140	150	70	156	212	412
**G15**	8	5	10	15	10	24	11	28	16	34	37	42	73	41
**G16**	16	1447	163	18	10	203	81	5	104	249	186	40	16	217
**G17**	18	538	121	22	30	117	61	11	80	160	174	40	37	147
**G18**	1548	1417	1960	1476	1847	1796	2911	5537	1284	1849	2159	1889	1919	1987
**G19**	22	18	32	23	29	75	48	43	61	74	88	66	112	52
**G20**	34	62	79	51	66	113	66	100	121	117	165	160	196	146
**G21**	171	753	544	151	144	418	596	235	389	553	806	581	403	786
**G22**	13949	12227	14063	5595	10035	12230	8809	34842	4429	5960	16343	7811	7756	8699
**G23**	29043	30665	30033	25711	20916	25732	39066	17992	33045	32703	27451	34732	25026	28307
**G24**	72	469	372	178	253	202	259	94	123	294	202	186	281	429
**G25**	67	930	284	120	169	111	161	98	54	167	169	110	98	218
**G26**	8	77	46	17	22	16	26	11	9	32	30	12	23	58
**G27**	761	2231	2193	1179	1570	992	1679	847	687	1248	1201	1133	1209	1630
**G28**	2231	9343	5180	7582	2124	3397	9165	535	7701	11790	2896	6666	3268	6809
**G29**	57	484	289	162	174	147	173	70	69	219	132	118	177	310
**G30**	42823	47892	54149	37468	38218	40703	52351	33740	44279	48832	47554	50147	45454	54016
**G31**	165	526	582	247	225	488	1005	323	487	836	1793	1139	774	1047
**G32**	35	171	113	77	72	116	125	29	68	134	175	89	143	135
**G33**	2023	1334	1199	3316	1910	1915	1632	982	1942	1813	796	1101	1175	885
**G34**	295	941	787	476	489	622	1772	333	414	813	1339	781	1003	989
**Sum**	248404	238803	247745	242912	248413	246637	241516	258307	243421	240595	251843	245341	247672	246063

Table S2.4: Absolute cell numbers in gates from G1 to G34 of samples picked between 79 and 172.5 h

Time	79 h	91 h	93 h	95 h	97 h	99 h	101 h	169 h	170 h	170.5 h	171 h	171.5 h	172 h	172.5 h
**G1**	9506	24449	27785	20746	42056	57005	43646	2599	2738	3874	3844	5487	4174	2685
**G2**	1968	1423	2481	1899	4952	8806	14034	9311	9417	10487	10501	16250	12061	8175
**G3**	1036	397	438	701	1867	3519	11054	13327	10103	11857	10868	11338	4360	6918
**G4**	421	284	409	479	805	1300	230	271	378	409	720	279	478	347
**G5**	50008	61942	54806	54521	44959	40584	32954	793	991	1198	1588	323	677	744
**G6**	53744	51129	35329	44504	33470	27706	21818	367	526	575	788	199	553	358
**G7**	2501	987	1370	1204	982	550	301	122	278	232	340	161	382	111
**G8**	1468	238	704	241	1877	1318	1596	2245	5991	4981	6021	3436	4861	3952
**G9**	299	144	1613	394	1276	721	720	1532	2458	2336	3332	1963	2324	2472
**G10**	631	7520	7369	11792	12308	6934	12160	13653	14957	15869	16388	11309	15463	17364
**G11**	95	1755	1790	642	1155	1034	441	227	308	419	496	388	696	447
**G12**	298	929	1902	1003	1115	1963	274	2289	2116	2210	2192	3140	3592	2436
**G13**	231	93	114	251	2077	7513	17024	28013	23810	24928	23741	27296	15618	26719
**G14**	484	392	749	834	505	3536	799	2492	2901	3713	2892	13263	4107	5222
**G15**	69	29	18	80	659	3601	11133	15486	14582	13486	14276	10241	11724	11965
**G16**	279	97	337	192	254	327	123	13460	2973	2507	2765	1303	2021	1603
**G17**	140	370	510	774	703	725	109	7770	2140	1681	1827	1276	1184	910
**G18**	1346	2062	1524	2484	1834	1300	501	116	247	248	337	131	426	134
**G19**	90	327	257	578	658	548	156	1611	1275	753	1169	432	777	644
**G20**	145	576	537	982	1331	1267	508	4396	3513	2710	3281	1742	2885	2238
**G21**	670	2126	2784	3886	4048	4479	1372	19496	17374	14859	13919	14500	17998	14124
**G22**	13210	15176	5857	19507	11593	3363	9012	237	1713	1782	715	95	618	182
**G23**	36663	26033	24341	27890	22383	12272	17675	380	2293	2844	966	132	591	303
**G24**	764	156	220	156	164	108	489	76	317	294	402	239	86	39
**G25**	548	56	122	44	81	19	89	32	1005	850	279	80	103	22
**G26**	126	10	29	16	55	11	158	33	796	705	281	371	61	19
**G27**	3079	489	900	690	511	166	192	81	581	502	296	75	216	78
**G28**	7234	1824	12545	1876	3855	4489	3680	1256	2849	3590	4157	447	1612	1512
**G29**	509	68	150	89	67	31	139	30	153	129	114	87	36	18

**G30**	55841	46663	50297	47514	41466	29359	30737	679	2581	3776	1847	253	955	601
**G31**	1263	1467	2000	2458	4007	6034	7287	66402	81836	80145	72767	104299	104191	101677
**G32**	383	84	91	110	329	556	1398	159	501	681	811	343	117	94
**G33**	273	246	440	357	584	1186	237	170	288	280	651	170	218	356
**G34**	1302	510	951	695	882	731	640	173	331	427	561	402	359	122
**Sum**	246624	250051	240769	249589	244868	233061	242686	209284	214320	215337	205132	231450	215524	214591

Table S2.5: Absolute cell numbers in gates from G1 to G34 of samples picked between 173 and 199 h

Time	173 h	173.5 h	174 h	174.5 h	175 h	175.5 h	176 h	187 h	189 h	191 h	193 h	195 h	197 h	199 h
**G1**	3361	3173	1450	2343	760	1305	2257	1210	1136	945	1347	1161	1710	2047
**G2**	4010	2719	912	1514	383	375	1044	348	534	423	771	799	681	1197
**G3**	1574	1000	465	969	214	223	774	239	363	347	641	763	398	674
**G4**	141	226	87	123	82	69	91	65	146	96	146	232	136	59
**G5**	286	668	219	387	269	119	224	184	514	342	456	1099	351	176
**G6**	166	327	110	177	128	53	116	119	250	191	237	996	249	99
**G7**	77	99	28	99	33	48	164	60	61	113	108	121	207	66
**G8**	3223	3548	2129	3235	1754	1014	1292	2284	2410	1685	1409	878	2438	1798
**G9**	1527	2032	1215	1921	1102	593	1080	1203	905	609	1038	751	724	692
**G10**	18379	14463	11740	19154	10513	7433	9264	10506	10220	5141	10181	7118	6579	9953
**G11**	297	243	134	216	106	176	292	269	185	109	169	109	221	147
**G12**	3226	6185	9576	13285	7585	15956	6723	1310	1188	850	1325	876	1585	1821
**G13**	14826	7119	2583	970	247	136	311	165	238	144	165	198	135	149
**G14**	6666	4388	3370	6624	3049	2586	9046	4313	3610	1351	3347	1663	3085	5232
**G15**	29585	18357	15200	3558	1659	552	740	479	462	539	419	303	193	201
**G16**	1080	1195	840	1129	835	1476	977	114973	89035	127111	71631	92762	131781	129573
**G17**	772	1532	994	4160	3516	7629	9389	20773	26538	21805	33365	29363	24430	20554
**G18**	56	106	46	70	53	29	50	44	86	83	70	230	103	34
**G19**	1066	3850	1185	8212	2252	1580	10221	2563	3454	2707	7262	8828	6399	6521
**G20**	3213	7181	6780	14391	8998	6330	19037	9535	11306	11403	16573	14519	9751	7729
**G21**	22876	30616	83676	66729	149104	160715	104997	40411	42061	36509	32910	26296	16425	21309
**G22**	176	425	198	153	91	68	90	132	188	164	167	977	604	147
**G23**	173	628	285	270	154	83	127	160	285	280	267	623	1018	158
**G24**	57	53	24	254	14	66	796	81	34	28	125	78	85	22
**G25**	29	140	56	46	13	23	80	40	75	28	35	62	66	31
**G26**	30	61	40	200	12	38	364	18	17	12	26	20	37	6
**G27**	82	118	39	70	32	29	105	87	78	50	60	145	266	72
**G28**	732	2069	805	1491	860	400	901	748	1951	1248	2025	2557	1251	836
**G29**	19	30	9	44	10	13	106	30	23	15	26	50	44	15
**G30**	318	1024	526	517	331	177	301	203	534	409	517	918	460	204
**G31**	96684	86471	87569	46162	24205	12595	9277	1985	1749	1569	1228	693	819	878
**G32**	65	69	47	204	18	100	558	118	25	28	78	64	63	44
**G33**	103	229	66	148	93	40	99	68	144	98	114	182	115	52
**G34**	120	90	63	172	43	125	276	155	54	108	122	110	237	123
**Sum**	214995	200434	232466	198997	218518	222154	191169	214878	199859	216540	188360	195544	212646	212619

Table S2.6: Absolute cell numbers in gates from G1 to G34 of samples picked between 211 and 221 h

Time	211 h	213 h	215 h	216 h	216.5 h	217 h	217.5 h	218 h	218.5 h	219 h	219.5 h	220 h	220.5 h	221 h
**G1**	6095	3148	2658	1744	2493	2973	1642	2281	2139	1928	1714	1339	996	3143
**G2**	4768	3804	2029	751	1216	1164	670	1063	980	684	865	679	549	1731
**G3**	4836	6468	1312	599	868	823	470	748	798	604	747	551	512	1698
**G4**	413	370	136	381	268	222	190	185	168	390	166	167	149	701
**G5**	1447	2841	174	804	1130	317	255	238	345	1515	607	798	853	1854
**G6**	1334	2294	128	945	619	471	302	320	361	830	491	429	512	1170
**G7**	630	2483	69	1075	1073	1511	762	1135	1039	1127	2233	943	779	1548
**G8**	1974	1270	524	695	705	772	545	577	690	600	405	448	338	389
**G9**	1506	1127	1030	746	811	559	471	574	1157	574	743	512	365	694
**G10**	21804	8688	2059	3621	3242	5224	4229	4109	4971	8354	3391	5018	4700	3105
**G11**	415	205	196	294	220	236	160	186	255	178	129	164	91	310
**G12**	3547	1774	19724	6635	14058	8785	14906	8154	7087	3206	3187	2358	1319	2195
**G13**	6344	16317	7514	3181	3001	2537	2679	2159	1827	1147	836	975	818	933
**G14**	11332	2766	950	2049	1393	1992	1705	1447	1921	2602	1360	2307	1952	1661
**G15**	9066	16744	8639	10587	4349	5150	5059	4392	4276	4445	3342	4311	3483	1470
**G16**	52745	31448	25842	19472	17365	23536	21139	19665	17777	16560	14513	17852	16771	19698
**G17**	13474	6535	3203	5933	12542	9223	4914	9113	8752	9147	10860	10358	13396	16682
**G18**	374	695	62	467	384	379	276	309	322	324	548	268	229	761
**G19**	1507	2192	1559	4385	3147	9055	2557	11515	10966	12792	2531	10886	12279	4409
**G20**	2647	3747	4847	12706	8271	18972	7987	20529	20472	22759	11801	25066	29029	13279
**G21**	9305	8163	59137	74156	80337	61916	101480	67235	72804	72877	118235	93690	93356	91720
**G22**	5777	24996	279	2028	2341	750	640	287	541	362	2361	255	377	683
**G23**	3168	15142	419	1287	3342	416	446	174	361	332	274	117	89	753
**G24**	568	1380	28	322	269	230	139	228	216	214	2983	509	557	388
**G25**	991	1894	149	582	964	155	105	112	82	85	5495	706	958	404
**G26**	266	182	69	128	576	78	23	61	57	74	692	172	180	129
**G27**	2595	10954	51	1324	798	473	368	204	303	182	12318	926	1454	404
**G28**	1810	3202	510	768	3932	434	442	380	559	1658	482	386	304	5138
**G29**	622	2421	9	279	198	104	59	85	94	85	2015	280	370	222
**G30**	923	1628	419	553	3599	135	147	139	186	639	321	211	204	1451
**G31**	9211	18502	77156	50585	42269	34077	47169	32174	29909	17587	16327	15000	10609	9832
**G32**	527	348	47	190	184	122	52	98	121	165	673	233	324	284

**G33**	345	360	66	163	169	127	89	92	109	217	105	99	83	540
**G34**	592	1005	116	1006	1242	1969	955	1490	1377	1200	1066	910	660	1636
**Sum**	182958	205093	221110	210441	217375	194887	223032	191458	193022	185443	223816	198923	198645	191015

Table S2.7: Absolute cell numbers in gates from G1 to G34 of samples picked between 221.5 and 267 h

Time	221.5 h	222 h	223 h	236 h	238 h	240 h	242 h	244 h	246 h	259 h	261 h	263 h	265 h	267 h
**G1**	993	860	816	7002	4281	3011	2817	2612	2022	1214	1480	1208	1289	1129
**G2**	579	683	567	4206	2888	1854	2064	1744	1143	982	1103	1077	1156	1001
**G3**	571	671	517	7103	6185	4543	4625	4358	3477	4233	3827	3555	4022	4119
**G4**	133	114	90	729	1116	515	958	1696	322	419	325	405	437	225
**G5**	714	876	678	370	434	655	530	777	1232	2601	2334	2764	3441	2760
**G6**	399	506	381	1247	4457	2971	4392	7319	2964	3505	3250	3489	4634	3130
**G7**	1102	824	896	35427	23506	12945	14036	11805	7927	6155	6024	6708	7546	4998
**G8**	381	395	436	180	223	202	174	112	129	63	79	41	33	40
**G9**	302	419	434	113	122	108	124	70	73	57	60	21	44	29
**G10**	4188	4909	4524	686	477	505	540	379	284	148	178	125	151	84
**G11**	104	97	115	219	108	72	102	133	74	105	57	72	85	46
**G12**	1260	1118	1296	155	138	117	134	79	76	30	39	21	20	29
**G13**	682	881	920	4627	3705	2371	2745	2482	1619	2363	1831	1806	2120	1869
**G14**	1505	1704	2022	139	321	238	405	295	157	92	157	59	91	87
**G15**	2072	3605	2503	3500	3021	1775	2331	2219	1185	2205	1646	1673	1963	1434
**G16**	18763	18787	19872	3480	1678	802	691	200	366	33	71	13	27	36
**G17**	25345	15221	20179	2099	648	282	249	111	147	16	42	25	16	42
**G18**	279	140	164	7987	9074	4044	5544	5542	3029	2125	1813	1947	2225	1245
**G19**	11817	12903	13548	894	604	238	460	461	125	179	174	226	201	81
**G20**	27985	30603	29943	1331	996	542	733	627	282	333	359	414	445	198
**G21**	92762	91837	86112	1671	952	573	714	533	290	168	238	217	223	142
**G22**	244	100	195	13739	26378	27281	29603	52602	31496	28319	35520	26190	31323	41092
**G23**	97	78	126	126	280	650	292	939	796	1931	3284	721	1508	4759
**G24**	731	249	368	24506	20506	15387	18146	13315	11527	12085	10594	14410	13765	9308
**G25**	349	70	156	16916	23211	36336	26399	13788	36942	49179	44883	35329	41007	45685
**G26**	108	68	86	9707	9640	11232	8378	6595	10517	11409	11934	8110	8647	13982
**G27**	750	148	242	28692	55326	69661	80542	80218	82934	73914	65753	96435	82741	62953
**G28**	268	252	297	152	201	361	192	272	660	750	1224	801	924	1429
**G29**	286	88	144	6754	6520	10603	8443	9403	9119	8919	8372	10566	9340	6640
**G30**	151	200	241	56	79	221	42	64	291	326	752	298	390	1221
**G31**	7302	8812	8314	3655	2646	1795	2233	1992	1002	1103	1036	1185	1235	850
**G32**	299	197	252	8301	7304	6267	6287	6015	5823	5504	5934	5057	5536	6712
**G33**	91	53	66	271	443	404	501	638	463	826	600	918	920	332
**G34**	698	591	569	11743	5161	4109	3255	2971	3015	1526	2126	1585	1722	1574
**Sum**	203310	198059	197069	207783	222629	222670	228681	232366	221508	222817	217099	227471	229227	219261

Table S2.8: Absolute cell numbers in gates from G1 to G34 of samples picked between 333 and 341 h

Time	333 h	335 h	335.5 h	336 h	336.5 h	337 h	337.5 h	338 h	338.5 h	339 h	339.5 h	340 h	340.5 h	341 h
**G1**	3512	2235	2331	2630	2317	3046	3339	3698	3814	2751	2508	3413	3822	4363
**G2**	3526	2020	2065	2200	2062	2334	2845	2707	2541	2113	2219	2636	3092	2980
**G3**	5625	4440	4164	3877	4161	4075	4275	2768	2311	3025	3301	3294	4640	3189
**G4**	364	506	425	603	639	570	816	621	572	760	642	758	790	655
**G5**	13525	9184	11462	14431	11853	14611	12364	16891	20106	13170	13465	12426	13567	19403
**G6**	6068	4416	4659	7025	6698	10182	14666	11868	11180	11653	10026	12447	12717	12847
**G7**	10156	7657	7625	9729	9698	10644	12212	11119	9533	9490	8162	10097	12288	10852
**G8**	23	2	14	0	15	45	2	4	0	3	1	0	3	0
**G9**	14	3	7	7	14	54	5	10	2	12	2	5	4	2
**G10**	27	35	25	35	40	94	19	25	9	18	15	6	36	6
**G11**	28	41	40	76	49	38	43	40	39	33	31	26	50	34
**G12**	15	12	21	24	25	16	17	40	56	27	23	29	25	49
**G13**	2447	1692	1801	1701	1640	1831	1853	1018	692	1026	1125	1228	2217	1324
**G14**	16	17	27	21	29	45	14	15	9	12	10	13	22	21
**G15**	1247	862	1060	1023	916	1177	1046	445	239	403	436	526	1302	705
**G16**	0	0	4	9	4	17	2	3	1	5	2	2	9	2
**G17**	5	3	5	2	5	19	4	9	4	5	5	4	8	7
**G18**	1146	937	874	1078	1150	1594	2111	1257	1173	1377	1117	1792	2125	1573
**G19**	171	67	100	104	88	177	229	109	62	73	85	130	296	189
**G20**	470	194	250	318	219	346	478	264	143	203	201	283	648	449
**G21**	421	179	221	289	199	347	490	415	433	300	313	401	595	691
**G22**	38576	42591	42513	49195	41430	41103	40912	40494	41317	42150	41930	43914	38821	47052
**G23**	22790	25868	26542	29451	25859	24446	25217	22460	24878	25736	26113	27367	25718	32777
**G24**	11621	11009	10067	9680	11001	9661	11840	11849	11357	13553	12805	13778	13207	10182
**G25**	30561	36844	35154	24496	33335	30878	28978	18822	13014	35628	38620	32880	26934	9304
**G26**	7001	9518	8970	6044	8087	7966	6711	6463	6002	8493	9652	8078	7116	5407
**G27**	39712	36489	37543	39629	36472	42100	39439	43347	42357	35002	34210	36454	36175	36043
**G28**	5056	4766	5821	6431	6070	2283	2400	7204	8686	6497	6074	3406	3011	3965
**G29**	7373	7541	6923	6306	8062	6047	7115	7215	6335	7716	7124	6578	6039	5158
**G30**	5762	5190	5808	5689	5026	2576	2479	4511	7725	3702	4270	2702	2400	6880
**G31**	3020	1520	1703	2046	1600	2072	2751	2076	1811	1671	1825	2214	3095	2798
**G32**	8738	8344	7086	5994	7608	6284	7123	5807	5107	6375	6916	6578	6913	5137
**G33**	1347	1211	1245	1944	1600	2435	2399	2518	1914	2311	2188	2328	2491	2016
**G34**	3056	2376	2327	2776	2610	3109	3129	3423	3438	2675	2271	3370	3595	3980
**Sum**	233419	227769	228882	234863	230581	232222	237323	229515	226860	237968	237687	239163	233771	230040

Table S2.9: Absolute cell numbers in gates from G1 to G34 of samples picked between 355 and 408 h

Time	355 h	357 h	359 h	361 h	363 h	365 h	367 h	403 h	405 h	405.5 h	406 h	406.5 h	407.5 h	408 h
G1	4379	3034	3224	3143	4079	4004	4140	4309	4054	4704	4492	4131	5120	4559
G2	3496	2412	2438	2281	2455	2665	2686	2818	2350	2845	3047	2603	3619	2633
G3	2929	2643	2616	2350	1599	2107	1761	1413	921	1633	1517	1320	1945	769
G4	814	626	585	603	615	850	538	299	322	1071	535	654	956	668
G5	8629	12499	10668	10273	15490	12196	9130	6173	5033	7613	5717	5325	3582	6284
G6	7743	5373	6092	8659	5876	7449	6089	4521	3414	8345	4924	5307	5172	4538
G7	11168	6255	7686	8198	7816	8397	7977	8788	6696	8892	7548	7237	7997	8225
G8	7	12	5	4	1	15	3	0	1	4	2	8	3	1
G9	7	13	3	6	4	12	5	3	4	5	4	17	1	1
G10	50	57	24	34	17	45	13	13	12	22	8	38	6	12
G11	31	33	26	19	34	35	23	31	25	54	36	40	32	28
G12	34	41	23	25	45	55	29	25	15	52	27	30	21	42
G13	1148	773	830	828	482	659	581	434	251	566	517	409	645	221
G14	28	29	18	18	12	26	8	9	8	21	22	60	13	6
G15	594	255	274	335	126	246	223	166	84	287	195	140	283	88
G16	7	14	3	4	3	6	5	5	1	4	0	5	3	3
G17	7	11	9	8	5	17	1	4	8	35	10	24	5	15
G18	1747	1052	1207	1139	1162	1462	1094	1327	996	2076	1520	1348	1207	1249
G19	223	98	96	107	69	109	63	54	24	146	111	69	178	55
G20	469	183	191	225	148	212	156	182	76	367	297	179	370	179
G21	729	345	345	294	406	501	369	522	412	846	725	582	992	806
G22	47101	44953	43721	48953	47430	47304	54304	65801	56317	59129	59995	61121	56188	61607
G23	50668	54401	54804	57297	61928	61084	71244	65807	93597	58882	70932	70045	75544	80645
G24	11226	8778	9870	8830	7362	8366	7900	7370	5633	7408	7375	7344	8232	5714
G25	20310	22468	26220	21086	10209	18873	15078	10476	10825	11760	10992	11410	8206	10608
G26	3870	6984	6824	5448	4054	4384	3913	2482	2382	2571	2516	2624	1922	2169
G27	33238	27956	31326	31345	27658	25765	27082	25883	18601	25960	26187	26912	27365	23458
G28	1593	6309	4104	5577	6027	5998	3529	3302	2780	3813	1963	1888	1139	2087
G29	7603	5592	6458	6493	5087	6376	6060	8002	6115	8520	8199	8269	7703	7370
G30	2376	8222	4270	4005	10320	6634	3988	3366	3372	4598	3023	3574	1798	3190
G31	3292	1908	1730	1646	1680	2070	1910	1910	1582	2311	2463	1899	3286	2034
G32	5253	5696	5887	4909	3669	4535	4125	3147	2305	3550	3337	3151	3655	2087
G33	1995	2003	2037	2034	2093	2320	1965	340	482	753	691	665	860	1569
G34	3647	2603	3034	2922	3355	3476	3352	3971	3259	4603	4027	3709	4264	4014
Sum	236411	233631	236648	239098	231316	238253	239344	232953	231957	233446	232954	232137	232312	236934

Table S2.10: Absolute cell numbers in gates from G1 to G34 of samples picked between 408.5 and 435 h

Time	408.5 h	409 h	409.5 h	410 h	410.5 h	411 h	411.5 h	412 h	412.5 h	427 h	429 h	431 h	433 h	435 h
G1	3788	4743	4626	4865	2575	2432	3516	3524	2471	2755	3486	3019	4425	3089
G2	2182	3227	2634	2738	1228	1262	1488	1614	1257	1513	1881	1448	2410	1502
G3	1484	1349	1003	1399	1093	1203	870	1048	1091	1401	806	846	1705	861
G4	1473	1197	1200	3359	621	446	426	298	258	373	242	201	402	354
G5	5841	5531	6271	6117	7408	14382	11639	9373	9728	6614	8444	9728	6960	7378
G6	7192	5909	6054	7606	4181	6309	7989	5983	4734	5644	3264	4654	5349	4621
G7	7994	7740	8157	8749	6649	6789	9420	9275	6878	9593	6656	7954	11568	9886
G8	0	2	0	0	4	2	1	4	0	0	0	2	6	1
G9	2	5	6	2	5	1	3	3	1	1	2	1	0	8
G10	4	13	13	8	29	3	32	36	4	2	0	5	60	8
G11	24	34	29	28	16	17	18	9	7	7	10	2	12	9
G12	76	27	54	78	41	37	69	28	23	9	17	14	28	23
G13	571	440	291	494	338	382	293	437	379	554	217	280	648	319
G14	16	14	20	15	8	29	5	4	8	25	4	7	19	14
G15	298	162	111	185	127	152	124	211	172	312	61	126	246	169
G16	8	7	2	1	14	15	40	1	12	4	8	3	1	2
G17	44	21	20	23	17	12	24	5	15	3	4	2	3	4
G18	1566	1483	1433	2556	874	1087	1513	1062	766	1182	711	694	1312	969
G19	254	84	68	77	19	25	42	67	32	66	29	29	68	32
G20	521	240	179	208	57	74	143	154	57	141	54	95	158	113
G21	1135	775	790	836	260	262	363	316	201	155	207	197	467	242
G22	50276	69117	71333	63780	62063	42686	56834	64695	64373	82636	85862	75276	79441	83315
G23	89587	54990	69907	63070	66277	56141	67192	77556	70510	44474	56984	66381	59311	62795
G24	5629	6809	5101	5861	5855	5798	4805	4993	4917	8583	5075	5089	7196	5769
G25	11333	12473	10072	12683	9472	6328	4281	5628	6409	11664	9192	6654	6599	7401
G26	2913	3083	2536	3349	2633	2406	1533	1813	1847	4042	1970	1898	1994	2034
G27	21934	29864	26017	25155	20921	17925	19836	21985	19304	27659	19256	20514	19819	22526
G28	2413	2868	3159	4412	11170	11623	10057	5575	10327	4036	7372	3724	3433	3549
G29	5681	9215	5462	5280	5025	4381	3562	3502	3929	8821	4986	4364	5854	5476
G30	3048	3498	3596	3618	7885	29861	14129	7713	11898	2524	5986	9967	3310	3890
G31	2124	2662	2097	2422	672	832	1057	1199	716	1038	1015	940	1802	1056
G32	2928	3141	2177	3149	2690	2877	2026	2034	2219	3575	2063	1874	3338	2142
G33	1805	1026	1425	3677	1139	1146	1239	1030	970	1635	1351	1297	2636	1901
G34	3646	4035	3725	4124	2720	2916	3946	3921	2872	3500	2854	3238	4792	3788
Sum	237790	235784	239568	239924	224086	219841	228515	235096	228385	234541	230069	230523	235372	235246

S2.6: Movie snapshots on AMC and CMC dynamics in the continuous reactor

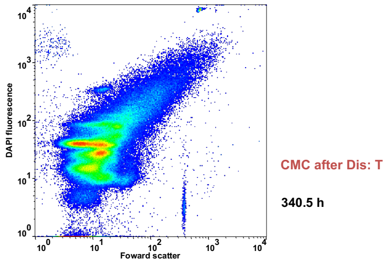

Figure S2.4: Microbial community described by its single-cell characteristics (FSC and DAPI fluorescence) in time series. Long-term and short-term pulse disturbances are marked on the right side of the 2D-dot plots. The full movie is provided as a QuickTime movie file (Movie S1.mov).

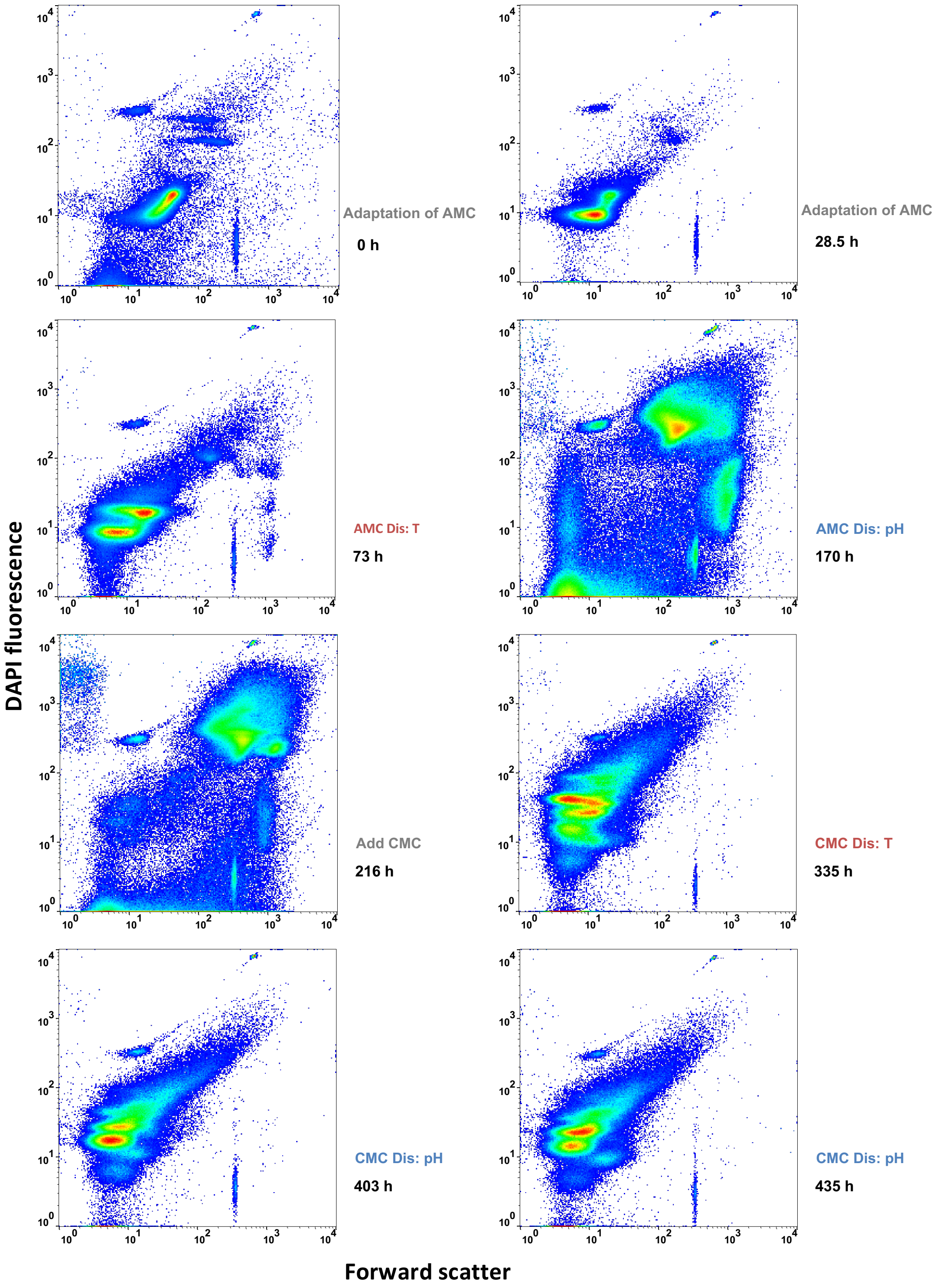

Figure S2.5: Exemplary 2D-dot plots (FSC and DAPI fluorescence) are shown. The samples were chosen according to the different disturbances addressed to the AMC and CMC in the continuous reactor.

S2.7: Variation in cytometric structures analysed by the flowCyBar

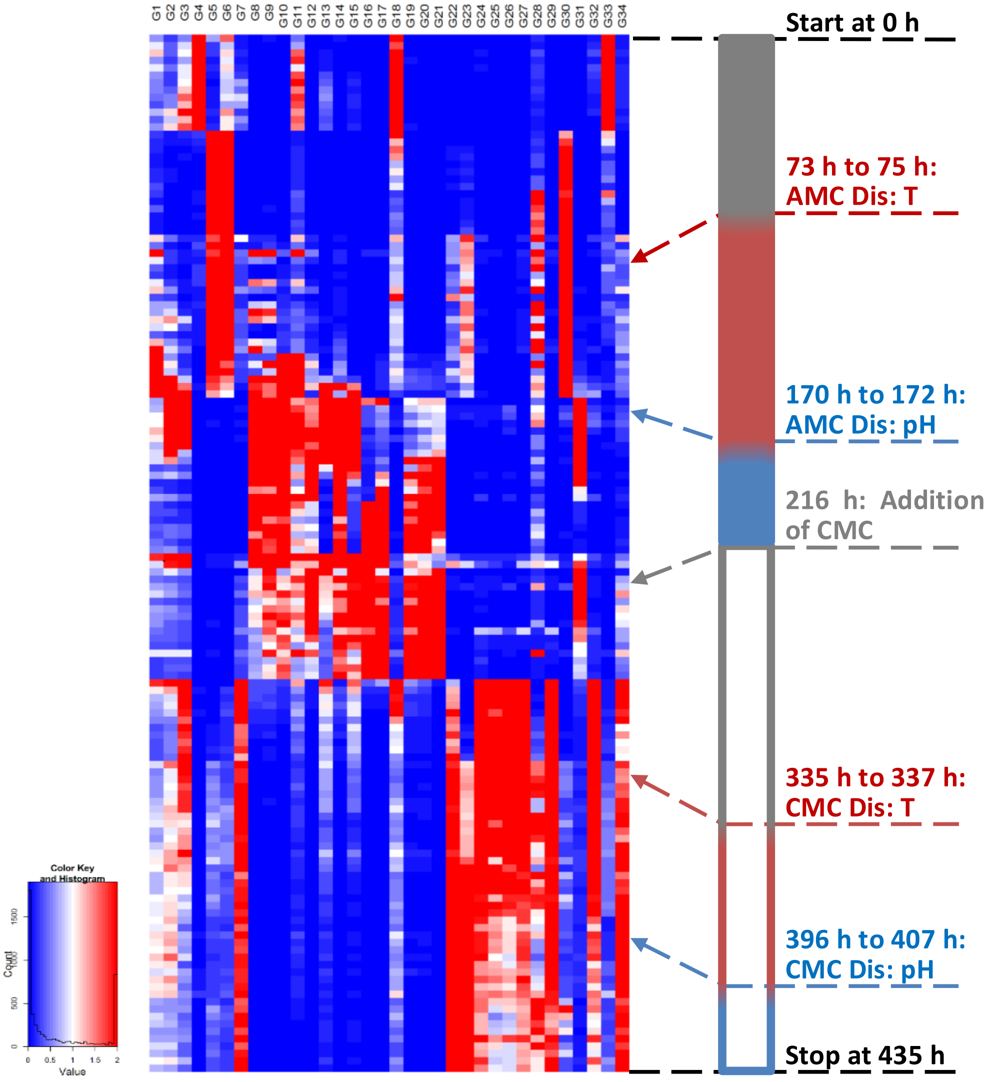

Figure S2.6: Changes in the cytometric community structure were visualized by using the FlowCybar tool (https://bioconductor.org/packages/release/bioc/html/flowCyBar.html). The relative cell abundance per gate (34 gates) is shown for each sampling point (140) during 435 h of cultivation (abundances given in Section S2.5). The scale of abundance is indicated by a color gradient, from the dark blue corresponding to low virtual cell abundance to red for high virtual cell abundance per gate (see colour key). The white color indicates the average of virtual cells per gate. Start of the various disturbances are marked by arrows (red for temperature disturbance, blue for pH disturbance and grey for the inoculation of the CMC) while the time periods after disturbances are outlined by a colour bar (right side) that corresponds to Figure 1.

It is obvious that the AMC showed low numbers of occupied gates with only a few gates above average comprising 4.38 % to 48.84 % of all cells at 0 h. The simple structure of the AMC varied during continuous cultivation due to alterations in the strains' dominances (up to 216 h). A complete structure shift, induced by CMC, was finally seen after 236 h. The addition of the CMC to the reactor (at 216 h), increased the cell number by at least one order of magnitude within the following 23 h (Fig. S1.1 in Text S1 in the supplemental material). As could be expected, the AMC was eventually overgrown by the CMC and vanished completely thus representing zero resilience. Also the sequencing data did not show any OTUs from the AMC despite a 1 % appearance in the G5 of the 412 h sample (*Comamonadaceae,* Fig. S5.6 in Text S5 in the supplemental material). Instead the CMC mock community (used as a positive control in the sequencing analysis) represented eight other OTUs and even most of the sorted gates did not contain any of the AMC members (Text S5 in the supplemental material, Section S5.4).

References

Koch, C., Harnisch, F., Schröder, U. & Müller, S. (2014) Cytometric fingerprints: evaluation of new tools for analyzing microbial community dynamics. *Frontiers in Microbiology*, 5, 273.
