# Supplementary material for: Ecological Stability Properties of Microbial Communities Assessed by Flow Cytometry
Source: mSphere. 2018 Jan 17;3(1):e00564-17. doi: 10.1128/mSphere.00564-17 (PMC5770544; doi:10.1128/mSphere.00564-17)
Supplement: TEXT S3 [file sph001182449s3.pdf]

Ecological stability properties of microbial communities assessed by flow cytometry

Zishu Liu[1], Nicolas Cichocki[1], Fabian Bonk, Susanne Günther, Florian Schattenberg, Hauke Harms, Florian Centler, Susann Müller

UFZ – Helmholtz Centre for Environmental Research, Department of Environmental Microbiology, Permoserstr. 15, 04318 Leipzig, Germany.

[1]divided first authorship

Supplemental Text S3

S3: Calculations of gate-based deviations in community structures over time

Figure S3.1 explains the procedure which is used to compute gate-based structural deviation of two different community states (state 1 and state 2). These two communities are schematically represented by the 2D-dot plots in Figure S3.1, A. Three gates (G1, G2, and G3) were defined which formed the gate-template for both 2D-dot plots, and the relative abundances of cell numbers per gate and per sample were determined. Following, states 1 and 2 are regarded as two points in a three-dimensional space (Fig. S3.1, B), whose coordinates are the relative abundance per gate per sample. The deviation between these two community states can be quantified as the distance (using Euclidean or Canberra distance) between these two points.

For our example experiment (Text S1 in the supplemental material), a 34-gate-template (Text S2 in the supplemental material, Section S2.4) was used for analyzing both AMC and CMC structures (in total 140 samples, Text S2 in the supplemental material, Section S2.7). If all 140 samples would be compared for determination of stability properties there would be 140 points in an 34-dimensional space. The deviations among points are calculated using methods described in Table 1 in the main text (Canberra distance) which were then used to quantify stability properties of both the AMC and CMC.

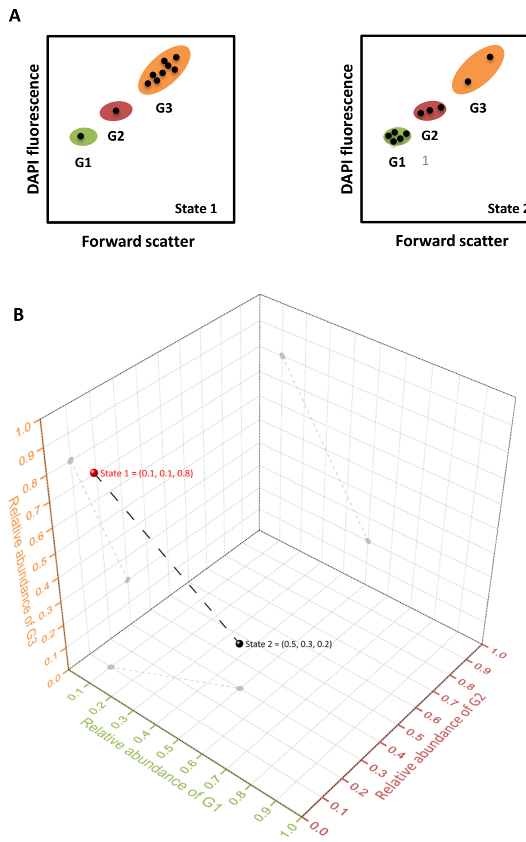

Figure S3.1: Workflow for computing gate-based structural deviation between two community states. **(A):** Schemes of two community structures described by 2D-dot plots. Gates G1 (green), G2 (red) and G3 (orange) were defined according to clusters containing cells with similar characteristics. **(B):** Relative abundances per gate and per sample were used to calculate two colored points in a three-dimesional space (red for state 1 and black for state 2). The distance (here using Euclidean distance, black line) can be used as the quantified deviation between these two community states.
