# Supplementary material for: Ecological Stability Properties of Microbial Communities Assessed by Flow Cytometry
Source: mSphere. 2018 Jan 17;3(1):e00564-17. doi: 10.1128/mSphere.00564-17 (PMC5770544; doi:10.1128/mSphere.00564-17)
Supplement: TEXT S4 [file sph001182449s4.pdf]

Ecological stability properties of microbial communities assessed by flow cytometry

Zishu Liu[1], Nicolas Cichocki[1], Fabian Bonk, Susanne Günther, Florian Schattenberg, Hauke Harms, Florian Centler, Susann Müller

UFZ – Helmholtz Centre for Environmental Research, Department of Environmental Microbiology, Permoserstr. 15, 04318 Leipzig, Germany.

[1]divided first authorship

Supplemental Text S4

S4: R script for calculation of stability properties

We provide an easy to use R script for the automatic calculation and visualization of stability properties based on flowcytometric data. The script can be obtained at GitHub under the URL https://github.com/fcentler/EcologicalStabilityPropertiesComputation. As input, the script requires a space- or tab-delimited text data file containing the evolution of relative gate population abundances over time. The first column is expected to contain time information, while the remaining *n* columns contain the evolution of the *n* gate populations. The file must contain a header line. At the beginning of the script, some input must be provided (see also comments within the script):

1. The filename of the data file must be provided.
2. If more than one experiment is stored in the data file, the user can select the start and end time of the experiment to be analyzed.
3. The time of the disturbance t_{ref} can be specified. If no t_{ref} is given, the script assumes that the first time points belong to the constant phase prior to the disturbance event. The first deviation from this constant phase is then identified by cluster analysis. The cluster containing the first time point of the experiment is selected, and the largest time point which belongs to this cluster is used as t_{ref}. The corresponding cluster dendogram is shown in the output. Note that this procedure only produces reasonable results if the disturbance leads to a noticeable change in community structure which is already recognizable at the sampling time directly succeeding the

time point of the disturbance.

4. The timepoint of maximal deviation from the reference state (t_{max}) can be given. If not specified, it will be computed automatically.

The script produces a PDF file as output, containing the following information:

1. In case t_{ref} is estimated by the script, the corresponding cluster dendogram is shown.
2. The evolution of gate abundances is visualized in three plots. In each plot, gray open circles indicate time points from the initial constant phase and black circles connected by lines indicate the evolution of the system after the disturbance event, with an arrow pointing to the first post-disturbance state. Filled symbols indicate the last time point of the experiment (state s_{end}). A red circle indicates the reference state s_{ref}. Triangle symbols indicate the state s_{max} most distant from the reference state based on Euclidean distance (blue) and Canberra distance (red). The three plots are:
    a. A NMDS plot visualizing the evolution of the entire system through time.
    b. Two plots showing the evolution of the system with respect to the two most abundant gates at the beginning and the end of the experiment (states s_{ref} ands_{end}). A circle centered at the reference state visualizes the reference space with radius $r_e \cdot \sqrt{2}$. Alternatively, the user can select other gate combinations to be plotted in these two plots. These can be specified in the beginning of the script.
3. The deviation from the reference state is plotted over time, using the Euclidean and the Canberra distance. Vertical lines indicate the limit of the reference space, using as y-coordinate r_e and r_c. Values for resistance RS and displacement speed DS are given in the legend.
4. Resilience RL is plotted over time for both Euclidean and Canberra distance. Values for RL and elasticity E for the last data point are given in the legend.
5. Resilience in online mode is plotted, using the Canberra distance.

As an example, Figure S4.1 shows the script output for evaluating the dataset 91 - 199 h which includes the Dis: pH experiment for the AMC. While the reference state was set manually before (Fig. 3 in main text), here, for demonstration purposes, we let the script automatically estimate t_{ref}.

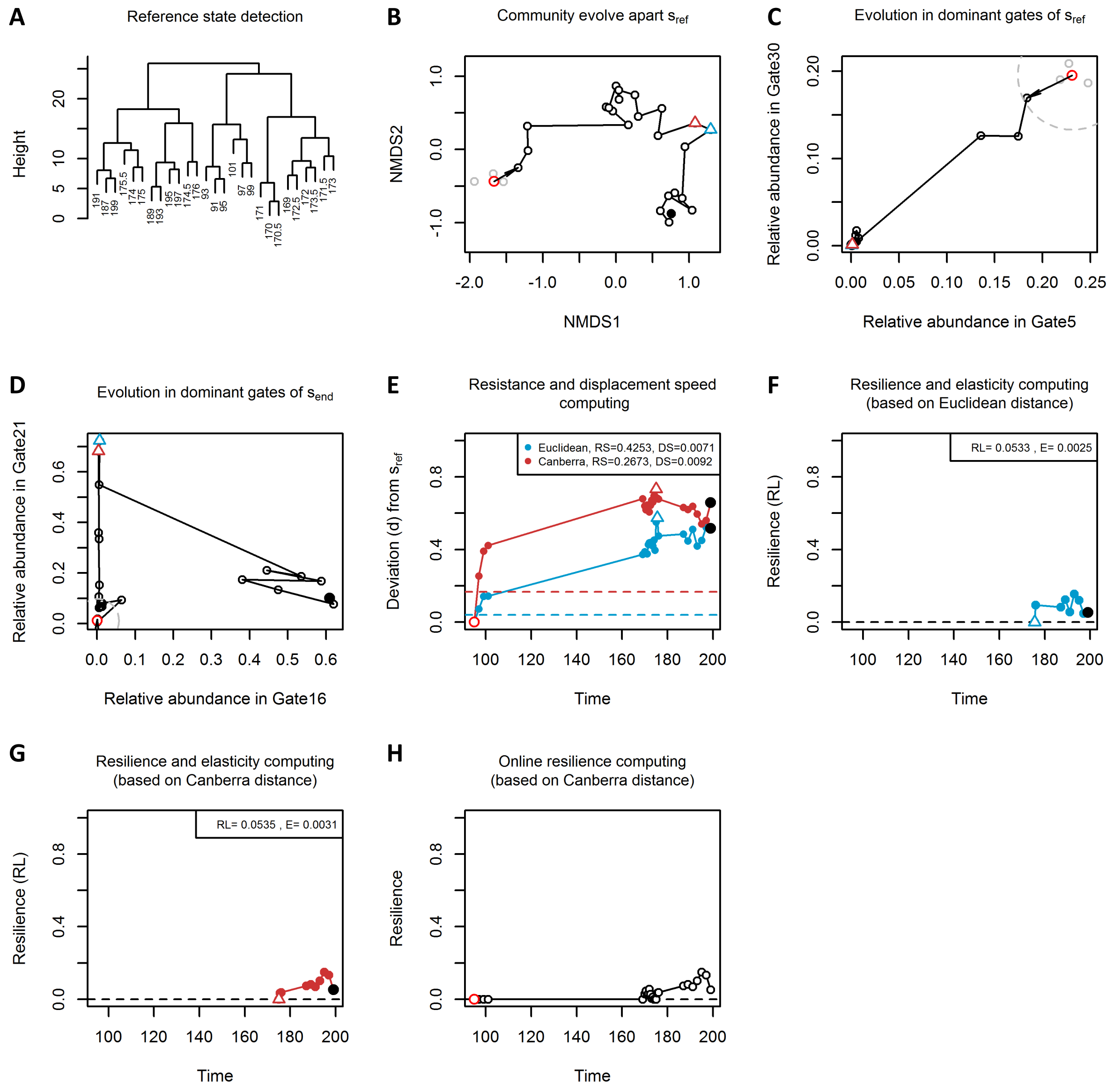

Figure S4.1: Post-hoc and *on-line* computation of the stability properties. Test samples were used from the time period of 91 h until 199 h (pH disturbance in the AMC). **(A):** A cluster dendogram was drawn to automatically compute the reference state s_{ref} (here defined by three time points: 91 h, 93 h and 95 h) and t_{ref} at 95 h. The evolution of gate abundances is visualized in three plots. **(B):** An nMDS plot for the whole microbial community (AMC). **(C):** The evolution of the two gates which had their highest cell abundances at s_{ref}. **(D):** The evolution of the two gates which had their highest cell abundances at s_{end}. In each plot, grey circles indicate the time points chosen to determine s_{ref} (91 h, 93 h, and 95 h) and black circles connected by lines indicate the evolution of the respective gate community. Filled symbol indicate the end of the experiment (s_{end}). Red circle indicates the reference state s_{ref}. Dashed circle marks the reference space. A black arrow marks the departure from the reference state. The triangle indicates s_{max} which has maximal deviation from s_{ref} (blue for Euclidean distance and red for Canberra distance). **(E):** Quantified structural deviation (see S3) from s_{ref} is plotted until s_{end}, using both Euclidean and Canberra

distance. According to our method (Table 2), resistence (RS) and displacement speed (DS) are computed. Dashed horizontal lines mark the limits of the reference space, given by r_e and r_c. **(F, G):** Resilience (RL) and elasticity (E) were computed based on Euclidean distance and Canberra distance respectively and plotted from t_{max} to t_{end}. **(H):** RL was computed in *on-line* mode. When ongoing variations in community structure lead to increases beyond previously encountered d_{max}, resilience is evaluated to be zero. Positive values indicate the onset of recovery.
