# Supplementary material for: Ecological Stability Properties of Microbial Communities Assessed by Flow Cytometry
Source: mSphere. 2018 Jan 17;3(1):e00564-17. doi: 10.1128/mSphere.00564-17 (PMC5770544; doi:10.1128/mSphere.00564-17)
Supplement: TEXT S5 [file sph001182449s5.pdf]

Ecological stability properties of microbial communities assessed by flow cytometry

Zishu Liu[1], Nicolas Cichocki[1], Fabian Bonk, Susanne Günther, Florian Schattenberg, Hauke Harms, Florian Centler, Susann Müller

UFZ – Helmholtz Centre for Environmental Research, Department of Environmental Microbiology, Permoserstr. 15, 04318 Leipzig, Germany.

[1]divided first authorship

Supplemental Text S5

S5: Sequence-based analysis

S5.1: Sample information used for sequencing analyses

Illumina® 16S rRNA gene amplicon sequencing was done to verify flow cytometric data. Two species (*Rhodococcus sp.* RAH1 and *Pseudomonas putida* KT2440) were used as mock strains and the whole CMC (242 h sample from the continuous reactor experiment) used as CMC mock community. Illumina® sequencing found 1 OTU for the mock strains, respectively, and 8 OTUs for the CMC (threshold 0.8 %). The threshold was set according to the rarefaction curves of the artificial microbial community (AMC) data where the number of cultivated strains was three. The data are shown in Table S5.2.

In addition, the original inoculum and the pre-culture used as inoculum for the continuous reactor were sequenced, subsampled up to 13 104 reads, and found to represent 211 OTUs (threshold of 0.1 %) and 15 OTUs (threshold of 0. 8%) as well as 86 OTUs (threshold 0. 1 %) and 19 OTUs (threshold 0. 8%), respectively.

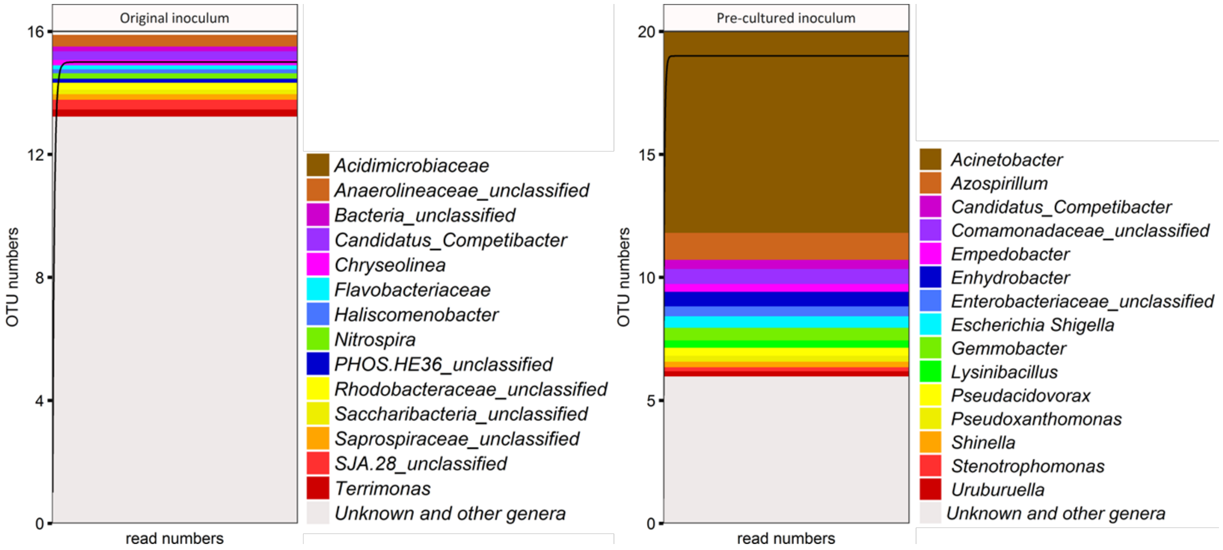

Figure S5.1: OTU rarefaction curves of original inoculum and pre-cultured inoculum at threshold 0.8 %. The original inoculum contained 15 known OTUs distributed over 14 genera while the pre-cultured inoculum contained 19 OTUs distributed over 15 genera.

Thirty samples in addition to the two mock strains were sequenced by Illumina® to a maximum of 50 000 reads per sample. This low number of reads was chosen because we were interested to verify the species richness in the AMC (which consists of only three organisms), in the CMC, and in the sorted gates of both microbial communities. To get a high resolution in diversity of samples was not the aim of this experiment. All samples (besides the mock strains) were subsampled to the minimum reads (2 631 reads, green line; maximum reads were 49 397) to enable for comparison of samples according to the workflow and testing of Props (Props et al. 2016). The rarefaction curves of all samples are presented in Figure S5.2 and the minimal amount of reads are marked by the green line (Fig. 5.2).

Table S5.1: List of the samples sorted (controls and gates) with their respective information; sampling time, gate number and the number of cleaned sequences obtained for each of them. The sample which has been taken as a reference for the normalization procedure is marked in grey.

Sampling time in h	Gate reference	Sequences per sample
4.5	G4	7 724
19	G5	6 217
19	G6	7 567
55	G5	9 325
55	G6	3 402
67	G1	8 530
67	G5	4 847
67	G6	6 804
67	G30	5 575
99	G1	9 024
99	G5	8 404
99	G6	5 167
99	G8	4 407
173	G15	40 742
173	G31	31 343
175	G21	35 240

Sampling time in h	Gate reference	Sequences per sample
199	G16	37 295
220	G10	4 161
220	G16	49 397
220	G20	30 315
220	G21	48 979
238	G24	2 631
238	G27	33 918
242	CMC mock	36 216
246	G25	39 518
246	G27	37 088
412	G5	26 793
412	G22	12 542
412	G23	16 618
412	G28	28 200
Mock 1		1 369
Mock 2		4 723

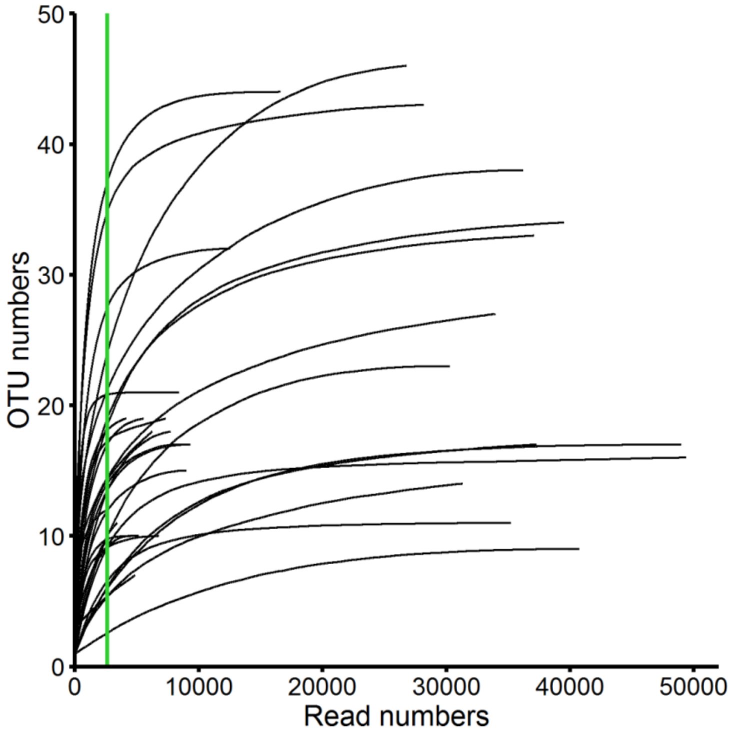

Figure S5.2: Rarefaction curves of 30 AMC and CMC samples. Subsampling to minimum reads was set to 2 631 reads.

The gates (Fig. S5.4) that were investigated by Illumina® sequencing were sorted using the sort option of the MoFlo by sorting 500 000 cells at a rate not higher than 2 500 particles per second and using the 'single-sort high-purity mode'. Figure S5.3 represents example gates which cover diverse numbers of OTUs that were phylogenetically affilitated by using the SILVA data base version 123. It needs to be mentioned that even by using the 'single-sort high-purity mode' (99 %) a sorting error of 1 % can be expected. Figure S5.3 clearly shows that some of the gates almost entirely comprise only one pure OTU.

Comamonadaceae	
G 4	97.2 %

Paenibacillus	
G 6	87.5 %

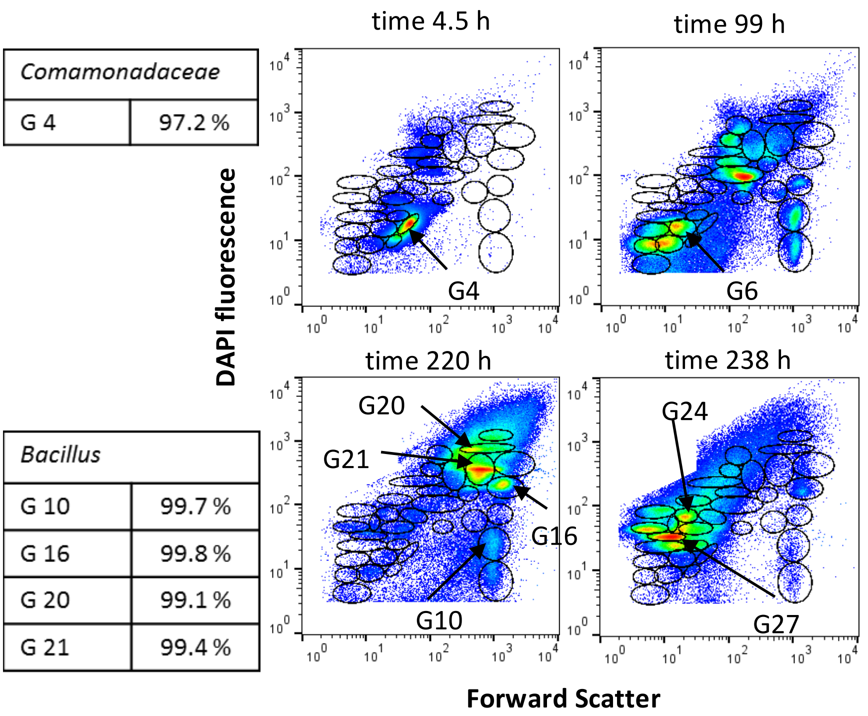

Bacillus	
G 10	99.7 %
G 16	99.8 %
G 20	99.1 %
G 21	99.4 %

Genus level	G 24	G 27
Providencia	88 %	72.1 %
Myroides	5.1 %	14.4 %
Proteus	3.1 %	0 %
Morganella	1.8 %	10 %
Rhodococcus	0 %	1.4 %

Figure S5.3: Exemplary 2D-dot plots for cell sorting: samples from the continuous reactor experiment were chosen cytometrically analyzed and sorted according to the gate-template (Text S2 in the supplemental material, Section S2.4). The sorted samples were frozen at -20 °C and further processed according to the Illumina® 16S rRNA gene amplicon sequencing protocol (see below). The tables in the figures mark the organisms and their respective sequence abundance in the gates of interest.

S5.2: DNA Extraction and quality testing

The DNA extracted from the sorted 500 000 cells following the Chelex protocol (Koch et al. 2013) was not enough in quantity to be detected by the *Qubit®* 3.0 for quality control (Life Technologies, Carlsbad, California, USA). Therefore a PCR step was performed to evaluate the quality of the isolated DNA by testing their amplified products by gel-electrophoresis. The PCR step was done with 35 cycles in a S1000 Thermal cycler (Biorad) by using the universal primers Forward 27F 5'-AGAGTTTGATCMTGGCTCAG-3' and Reverse 1492R 5'-TACGGYTACCTTGTTACGACTT-3' following the recommandations of Lane (1991).

S5.3: Library preparation for Illumina®

DNA directly isolated from sorted cells was used for the PCR reaction to prepare a library for the Illumina® sequencing. Primers were designed to amplify the V3-V4 regions of the bacterial 16S rRNA

gene region. Based on the Silva data base (Quast et al. 2013) *in silico* tests and results the following primers for procaryotes revealed a theoretical identification of 76.3 % of the sequences referenced in the data base (version 123, updated on 2015, september the 11th): Pro341F 5'-CCTACGGGNBGCASCAG-3' (Takahashi et al. 2014) and Pro805R 5'-GACTACNVGGGTATCTAATCC-3' (Herlemann et al. 2011). The dual-barcoding was performed with the following list of 6 bp-barcodes: GTGAGC, TCCGCT, ACAGTG, CTTGTA, GACTCT, TGACCA, ATCACG, CCGGAG, GGCTAC, TAGCTT, CAGATC, CGATGT for the construction of the 5'-Barcode-Primer-Forward-3' and CTCTCT, TGTCTA, GCCAAT, CGTTAA, GGTATA, ATTCGC, GAGGAT, TTGTTC, TTAGGC, AGCGAC for the 5'-Barcode-Primer-Reverse-3' according to the Fasteris protocol (Fasteris SA, Plan-les-Ouates, Switzerland).

The library was done according to the following PCR protocol: 1 µL DNA solution; 2 µL 10 nM barcoded forward and reverse primers (Eurofins Scientific, Luxembourg City, Luxembourg); 0.2 units of Phusion® High-Fidelity Polymerase (New England Biolabs, Ipswich, Massachusetts, USA); 2 µL 5 x Phusion® GC solution and, 0.8 µL $MgCl_2$ solution to adjust to a final concentration of 2 mM (both solutions from Phusion® GC and $MgCl_2$ were provided in the Phusion® High-Fidelity Polymerase kit). Further, 0.2 µL of 10 mM dNTP mix (Promega, Fitchburg, Wisconsin, USA), and the nuclease free water (Qiagen, Velno, Netherlands) were added for adjusting the final reaction volume to 10 µL. The PCR reactions started with 3 min incubation at 95 °C, 30 s at 95 °C, annealing for 60 s at 52 °C, elongation for 60 s at 72 °C. The final elongation step was performed for 5 min at 72 °C before cooling down to 4 °C. The first step PCR, run for 20 cycles, and the negative controls (without DNA), run for 35 cycles, were verified by gel electrophoresis (1.5 % agarose) to ensure that no contamination was amplified. All confirmed PCR reaction free of contamination were purified by Agencourt® AMPure® XP-Kit (Beckman Coulter, Brea, California, USA) following the recommended protocol, and resuspended in 10 µL of nuclease free water (Qiagen, Velno, Netherlands). The second step PCR with the barcoded-primers was done with the same conditions of the first step PCR for 8 cycles, then purified by Agencourt® AMPure® XP-Kit as well and quantified by *Qubit®* 3.0 (Life technologies, Carlsbad, Califorania, USA) using the HS DNA kit (Life technologies, Carlsbad, California, USA). To ensure that no contamination was amplified in the second step PCR, negative controls were run for 35 cycles and checked on gel as mentioned above. The amplified and barcoded PCR products were pooled to a final volume of 30 µL following the Fasteris recommendations. This pooled sample was designed to get information on the prominent organisms in samples or gates (50 000 reads per sample for the AMC and 200 000 reads for the CMC before cleaning merging and normalisation steps). Sequencing was done by the Fasteris company (Plan-les-Ouates, Switzerland) on an Illumina Miseq machine using the V3 kit, 2 x 300 bp (V3 kit, Illumina, San Diego, California, USA).

S5.4: Evaluation of sequence data

Regarding the average quality score of the sequencing data set (q = 27.25) the reduction of sequencing and PCR errors was done by merging the Forward and Reverse sequences before the library demultiplexing and cleaning procedure by using Mothur version 1.36 (Schloss et al. 2009). After removing chimeras by Uchime (Edgar et al. 2011) the remaining sequences were gathered into OTUs using Mothur's average neighbor clustering algorithm and a 97 % sequence similarity cut off. The rarefaction curves were drawn using ggplot2 package in R (Wickham 2009). The analysis was done from a sequencing set of 615 096 Forward-Reverse overlapped sequences. Mothur was also used for the normalisation procedure that enabled to compare samples of different read numbers (samples from AMC and CMC) which were subsampled to 2 631 reads (see also Section S5.1).

OTU classification was done in Mothur using the data base SILVA version 123 (Quast et al. 2013). Obtained rarefaction curves contained a certain number of OTUs per sample, when a plateau was reached for each of them. The two mock strains showed only one OTU, respectively, while the mock community ended up with eight OTUs under the conditions that 2 631 reads were used as part of the normalization procedure and a relatively high threshold of 0.8 % was chosen. This threshold was defined according to recommendations given by Bokulich (Bokulich et al. 2013) towards an adequate OTU abundance threshold. This threshold allowed us to detect the three OTUs for the samples belonging to the AMC and to define a maximum list of 31 different OTUs found in the bioreactor (435 h, Table S5.2).

Table S5.2: OTUs of the mock AMC (OTU 01 - 03) and the CMC (OTU 04 - 31) from the eighteen sorted gates with their abundances in the whole normalized sequencing data set.

OTU	Reads	Phylum	Genus
01	26 706	*Proteobacteria*	*Comamonadaceae*
02	25 203	*Firmicutes*	*Bacillus*
03	11 656	*Firmicutes*	*Paenibacillus*
04	7 632	*Proteobacteria*	*Providencia*
05	4 610	*Bacteroidetes*	*Myroides*
06	3 222	*Proteobacteria*	*Alcaligenes*
07	2 557	*Proteobacteria*	*Burkholderia*
08	2 337	*Proteobacteria*	*Brevundimonas*
09	2 132	*Proteobacteria*	*Pseudochrobactrum*
10	1 175	*Proteobacteria*	*Morganella*
11	260	*Proteobacteria*	*Enterobacteriaceae*
12	167	*Bacteroidetes*	*Myroides*
13	142	*Proteobacteria*	*Proteus*
14	108	*Bacteroidetes*	*Myroides*

15	98	*Proteobacteria*	*Mesorhizobium*
16	97	*Actinobacteria*	*Propionibacterium*
17	93	*Proteobacteria*	*Bradyrhizobium*
18	90	*Firmicutes*	*Staphylococcus*
19	86	*Bacteroidetes*	*Myroides*
20	71	*Actinobacteria*	*Rhodococcus*
21	63	*Proteobacteria*	*Brevundimonas*
22	61	*Proteobacteria*	*Sphingomonadaceae*
23	58	*Proteobacteria*	*Brucellaceae*
24	55	*Bacteroidetes*	*Hydrotalea*
25	51	*Actinobacteria*	*Corynebacterium_1*
26	51	*Bacteroidetes*	*Elizabethkingia*
27	38	*Proteobacteria*	*Pelomonas*
28	36	*Bacteroidetes*	*Flavobacterium*
29	36	*Bacteroidetes*	*Prevotella_9*
30	30	*Proteobacteria*	*Pandoraea*
31	29	*Proteobacteria*	*Comamonas*

The Illumina® sequencing data was used to verify the cytometric data. For the two mock strains only one OTU respectively was found while the whole CMC mock community was represented by eight most abundant OTUs which were present at the sampling time of 242 h. For a natural microbial community, OTU number expectation can be higher than what we found in this study. However, we choose a procedure that aimed at the determination of the most abundant instead of rare and unknown OTUs. The PCR reaction was set to only 28 cycles to avoid over-amplification of the abundant species and to exclude the rare biosphere which would need more cycles to appear in our analysis. In addition, samples were pooled and only a maximum of 50 000 - 200 000 reads per sample were sequenced. The OTU abundance threshold was set to 0.8 % in our application which is rather conservative (Degnan & Ochman 2012). The γ diversity of our experiment revealed 31 OTUs under the chosen boundaries indicating that cell sorting contributed to a higher resolved information on community composition.

Generated rarefaction curves confirmed one OTU each for the used mock strains 1 and 2 which correspond to *Rhodococcus sp*. RAH1 and *Pseudomonas putida* KT2440, respectively. These two mock strains were used as positive controls for the MiSeq sequencing run. In short: *Rhodococcus sp*. RAH1 was obtained from the strain collection of the Helmholtz Centre for Environmental Research (Germany) while *P. putida* KT2440 was obtained from the German Collection of Microorganisms and Cell Cultures (Germany). The strains were cultivated aerobically in batches of 100 mL Lysogeny broth medium in 500 mL flasks separately on a rotary shaker at 30 °C and 150 rpm.

Three OTUs were confirmed for the AMC by rarefaction curves. The AMC was constructed artificially and contained three strains detected on the genus level as *Bacillus* and *Paenibacillus*, as well on the family level as *Comamonadaceae* on the SILVA data base version 123 and cultivated as described in Text S1 in the supplemental material. Due to usually (Comas-Riu & Vives-Rego 2002) highly diverse physiological states of the chosen strains as much as 8 different gates could be defined for the cytometric fingerprint of the AMC. The AMC structure changed rapidly due to the highly dynamic evolution of various cell states which occupied even several gates in one 2D-plot with just one species (e.g. growing cells or spores for either *Comamonadaceae* or *Bacillus*: G1, G5 or G15, G31) with changing cell abundances (and species) per gate. The CMC did not show gates with exclusive organisms suggesting that the organisms of this community are more similar in phenotype. The rarefaction curves of the CMC confirmed all 31 OTUs from Table S5.2 and showed always several OTUs per gate.

Cell sorting was used to test for presence and location of organisms (Section S5.1). The sorting scheme is shown Figure S5.4.

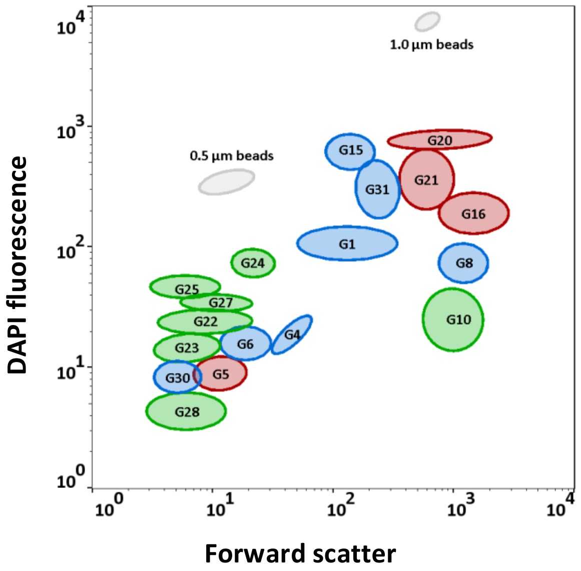

Figure S5.4: Representation of the gates which were sorted and sequenced. The corresponding blue gates were sorted for the AMC, the green gates were sorted for the CMC and the red gates were sorted both for the AMC and CMC.

The number of OTUs comprised between one and three, and some of the gates sorted from the AMC contained only one OTU, as expected (Fig. S5.5). For instance, AMC gates G1, G4 and G5 (up to 67 h of cultivation) contained only one OTU, respectively, while others contained two or three organisms at the same sampling time (e.g. G6, 55 h or G8, 99 h, respectively). These findings prove an identifiable position of the three organisms in the 2D-dot plots.

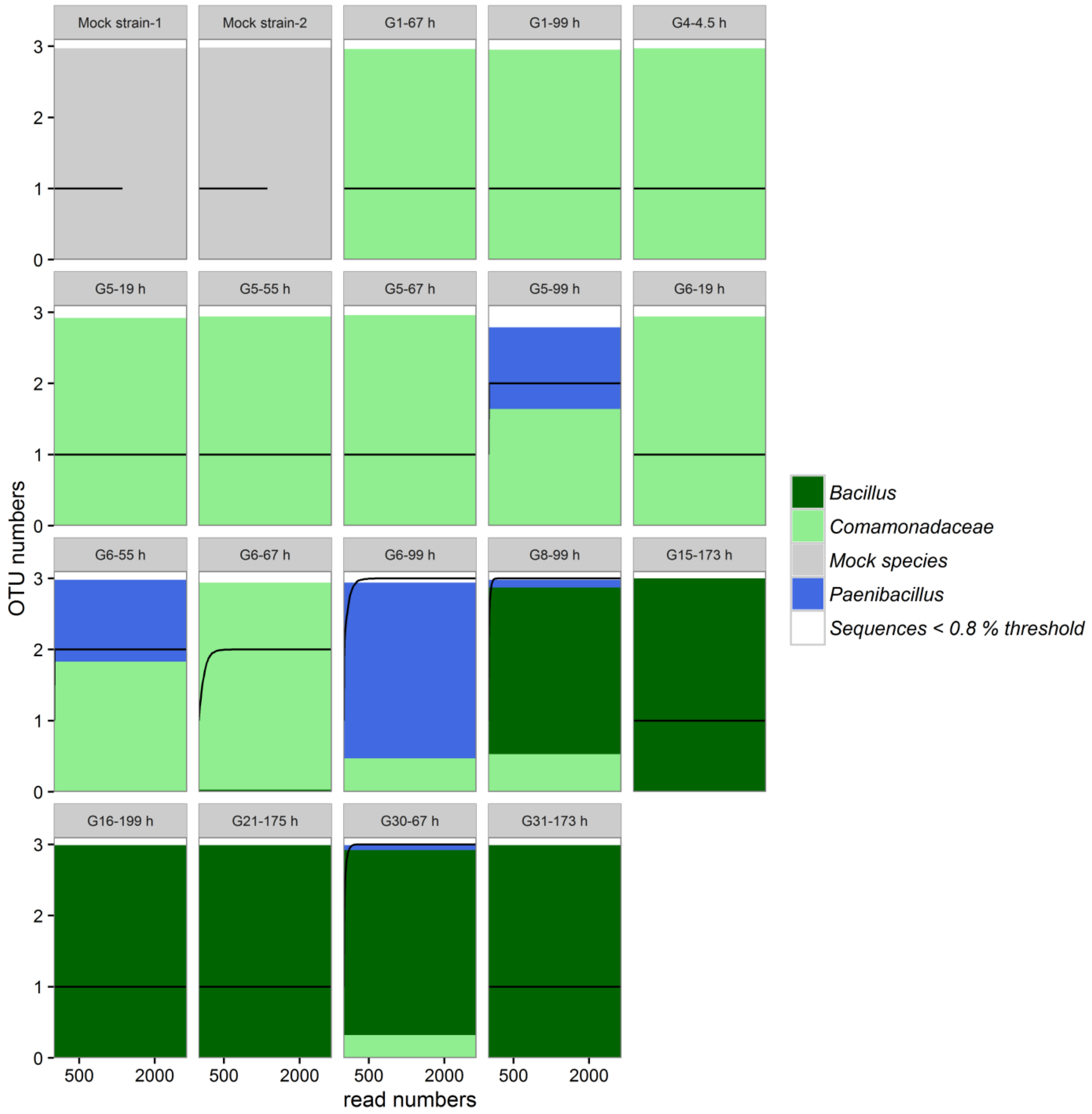

Figure S5.5: Rarefaction curves of two mock strains 1 and 2 (*Rhodococcus* sp. RAH1 and *Pseudomonas putida* KT2440) and sorted gates of the AMC. The light green colour represent the *Comamonadaceae*, dark green *Bacillus,* and blue *Paenibacillus*.

For the CMC mock community a maximum of eight OTUs were found above the defined 0.8 % threshold (Fig. S5.6). 12 gates were sorted and comprised between one and eight OTUs which are listed in Table S5.2. The data show that the establishment of the CMC (at 216 h) was not as prevailing as expected from the start because the *Bacillus* was still found dominant in gates G10, G16, G20, and G21 at 220 h. Later on, after 238 h very different organisms can be detected in the gates which are also different between

gates. The members of the original AMC were not present any more besides the very low abundancies of the *Comamonadaceae* (G5, 1.06 %) at the end of the continuous reactor experiment (412 h).

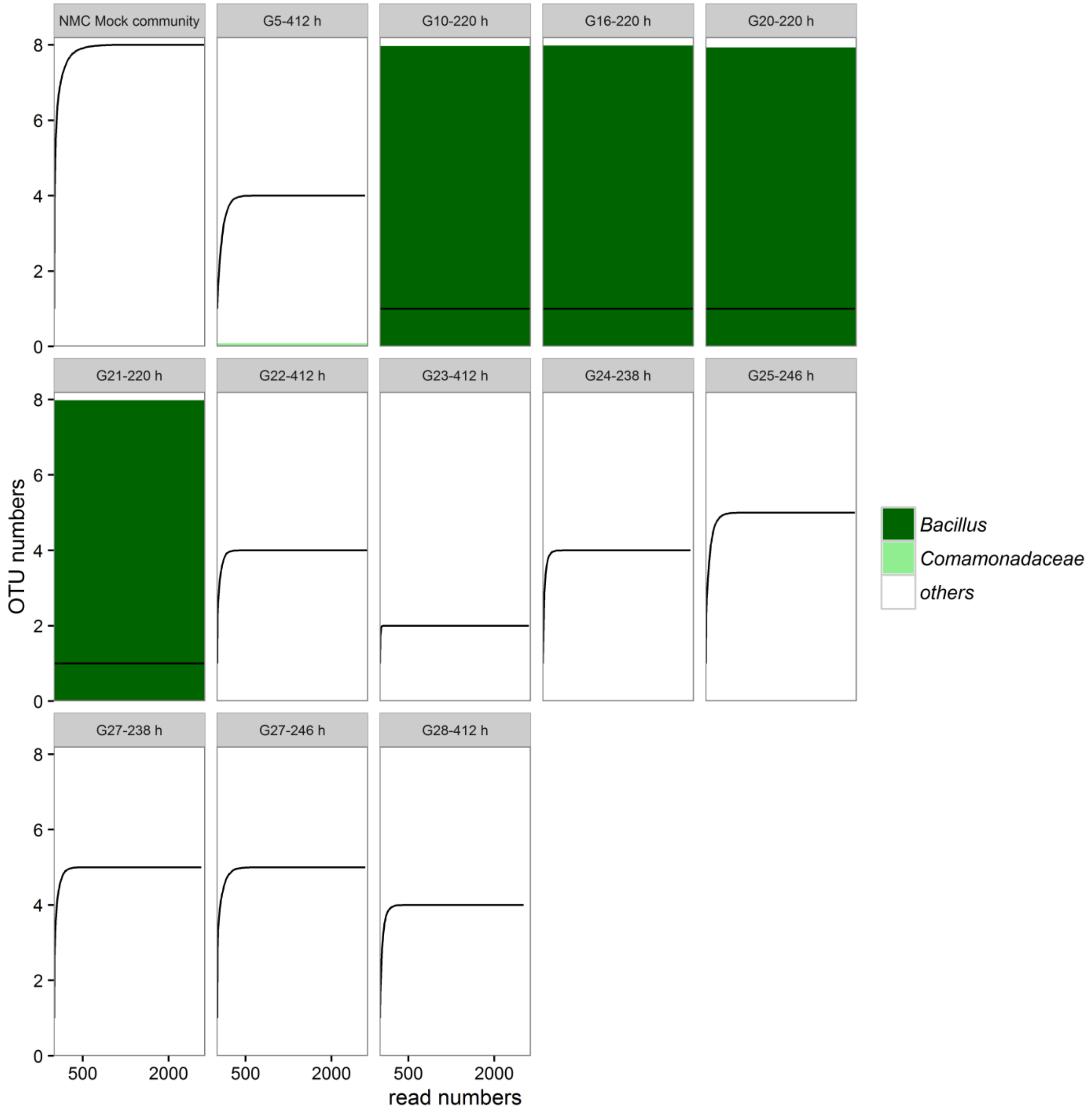

Figure S5.6: Rarefaction curves of the CMC mock community and sorted gates of the CMC. The light green colour represent the *Comamonadaceae* and dark green *Bacillus*.

S5.5: Influence of PFA and ethanol treatments on sequencing results

The influence of PFA stabilization and ethanol storage was tested using a wastewater sample. Only minor differences were found between fresh and PFA/ethanol fixed samples (Fig. S5.7).

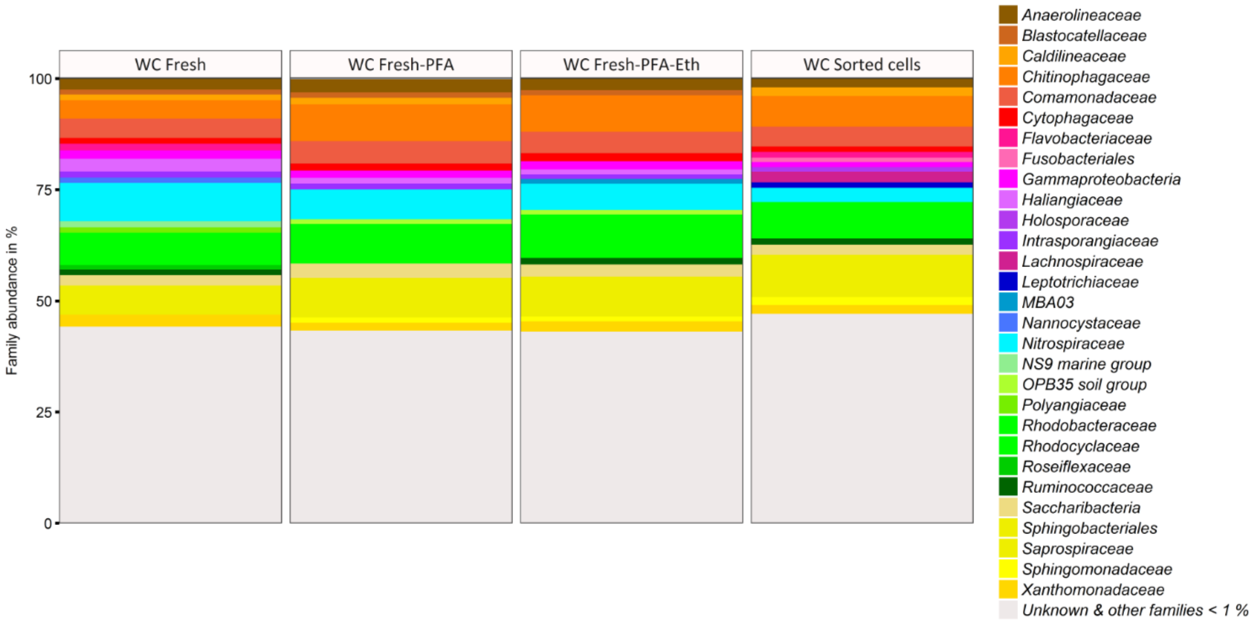

Figure S5.7: Community composition on family level (considering OTUs present at an abundancy above 1%, using 16S RNA gene sequences, region V3-V4) in samples WC Fresh (Whole community), WC Fresh-PFA (Whole community treated with PFA for 30 minutes), WC Fresh-PFA-Eth (Whole community, treated with PFA for 30 minutes and stored using Ethanol at -20 °C), WC Sorted cells (Whole community treated and sorted by Flowcytometry, 500.000 cells). The normalisation of the data was done on 45624 reads per sample.

References

Bokulich, N.A., Subramanian, S., Faith, J.J., Gevers, D., Gordon, J.I., Knight, R., Mills, D.A. & Caporaso, J.G. (2013) Quality-filtering vastly improves diversity estimates from Illumina amplicon sequencing. *Nature Methods,* 10, 57-59.

Comas-Riu, J. & Vives-Rego, J. (2002) Cytometric monitoring of growth, sporogenesis and spore cell sorting in *Paenibacillus polymyxa* (formerly *Bacillus polymyxa*). *Journal of Applied Microbiology* 92*,* 475-481*.*

Degnan, P. & Ochman, H. (2012) Illumina-based analysis of microbial community diversity. *ISME Journal,* 6, 183-194.

Edgar, R.C., Haas, B.J., Clemente, J.C., Quince, C. & Knight, R. (2011) UCHIME improves sensitivity and speed of chimera detection. *Bioinformatics,* 27, 2194-2200.

Herlemann, D.P., Labrenz, M., Jürgens, K., Bertilsson, S., Waniek, J.J. & Andersson, A.F. (2011) Transitions in bacterial communities along the 2000 km salinity gradient of the Baltic Sea. *ISME Journal*, 5, 1571-1579.

Koch, C., Günther, S., Desta, A.F., Hübschmann, T. & Müller, S. (2013) Cytometric fingerprinting for analysing microbial intra-community structure variation and identifying sub-community function. *Nature Protocols,* 8, 190-202.

Lane, D.J. (1991) 16S/23S rRNA sequencing. *Nucleic Acids Techniques in Bacterial Systematics* (eds Stackebrandt, E. & Goodfellow, M.), pp. 115-147. John Wiley & Sons, Chichester.

Props, R., Monsieurs, P., Mysara, M., Clement, L. & Boon , N. (2016) Measuring the biodiversity of microbial communities by flow cytometry. *Methods in Ecology and Evolution,* 7, 1376-1385.

Quast, C., Pruesse, E., Yilmaz, P., Gerken, J., Schweer, T., Yarza, P. , Peplies, J. & Glöckner, F.O. (2013) The SILVA ribosomal RNA gene database project: improved data processing and web-based tools. *Nucleic Acids Research,* 41, 590-596.

Schloss, P.D., Westcott, S.L., Ryabin, T., Hall, J.R., Hartmann, M., Hollister, E.B., Lesniewski, R.A., Oakley, B.B., Parks, D.H., Robinson, C.J., Sahl, J.W., Stres, B., Thallinger, G.G., Van Horn, D.J. & Weber, C.F. (2009) Introducing mothur: open-source, platform-independent, community-supported software for describing and comparing microbial communities. *Applied and Environmental Microbiology,* 75, 7537-7541.

Takahashi, S., Tomita, J., Nishioka, K., Hisada, T. & Nishijima, M. (2014) Development of a prokaryotic universal primer for simultaneous analysis of *Bacteria* and *Archaea* using next-generation sequencing. *PLoS ONE,* 9, e105592.

Wickham, H. (2009) *ggplot2: Elegant graphics for data analysis*. Springer-Verlag New York .
