# Supplementary material for: Ecological Stability Properties of Microbial Communities Assessed by Flow Cytometry
Source: mSphere. 2018 Jan 17;3(1):e00564-17. doi: 10.1128/mSphere.00564-17 (PMC5770544; doi:10.1128/mSphere.00564-17)
Supplement: TEXT S6 [file sph001182449s6.pdf]

Ecological stability properties of microbial communities assessed by flow cytometry

Zishu Liu[1], Nicolas Cichocki[1], Fabian Bonk, Susanne Günther, Florian Schattenberg, Hauke Harms, Florian Centler, Susann Müller

UFZ – Helmholtz Centre for Environmental Research, Department of Environmental Microbiology, Permoserstr. 15, 04318 Leipzig, Germany.
[1]divided first authorship

Supplemental Text S6

S6: Diversity metrics of cytometric data

In a recent study, Props et al. (2016), showed that microbial community diversity calculation based on single cell data can provide ecological information comparable to 16S rRNA amplicon sequencing. Cytometric α-diversity measures reveals the phenotypic complexity of a community and is a fast tool to follow community structure changes over time. Here, following Props's study, we used the approach to get an indication on the complexity of the AMC and CMC, respectively. For example, we analysed the diversity of two samples of the AMC and CMC, respectively, before the onset of the temperature disturbance (71 h, 333 h; Text S1 in the supplemental material). The diversities D_q were determined using order-based Hill numbers (Hill 1973) and named "phenotypic diversity". In contrast to the 128 X 128 binning grid used by Props et al. (2016), we used a gate-template with 34 gates (Text S2 in the supplemental material, Section S2.4). Table S6.1 shows the resulting "phenotypic diversities" for D_0 (q = 0), D_1 (q = 1) and D_2 (q = 2) for both the AMC and the CMC. When q = 0, only richness is considered. By increasing q values, additional emphasis is placed on the actual distribution of cells over the gates (relative gate abundances). For both time points, D_0 can reach a high value (34 and 33, respectively). This might be due to random effects because even a gate with only one event will be counted as one "phenotype". To avoid this problem, we introduced the average fraction of cells per gate of 2.94 % for D_0 (see main text section "Cytometric evaluation tools") as a threshold to count a gate as a "phenotype". For D_0 (with threshold), D_1 and D_2 the CMC showed always a higher diversity than the AMC. Still, the

values for the AMC were atypically high which, however, might be caused by the variable phenotypic complexity of its members (discussed in Text S5 in the supplemental material, Section S5.4). These data indicate higher diversity for the CMC which may be one reason why this community showed higher stability properties such as resilience and resitance in our experiment. Many studies discussed similar findings (Hooper et al. 2005).

Table S6.1: Phenotypic diversity based on single cell data for AMC and CMC states before any disturbances

States	Time points	D_0	D_1	D_2	D_0 (treshhold 2.94 %)
AMC before temperature disturbance	71h	34	9.55	6.62	8
CMC before temperature disturbance	333h	33	14.23	10.29	11

References

Hill, M.O. (1973). Diversity and evenness: a unifying notation and its consequences. *Ecology*, 54, 427-432.

Hooper, D.U., Chapin, F.S., Ewel, J.J., Hector, A., Inchausti, P., Lavorel, S., Lawton, J.H., Lodge, D.M., Loreau, M., Naeem, S., Schmid, B., Setälä, H., Symstad, A.J., Vandermeer, J. & Wardle, D.A. (2005) Effects of biodiversity on ecosystem functioning: a consensus of current knowledge. *Ecological Monographs*, 75, 3-35.

Props, R., Monsieurs, P., Mysara, M., Clement, L. & Boon , N. (2016) Measuring the biodiversity of microbial communities by flow cytometry. *Methods in Ecology and Evolution,* 7, 1376-1385.
